# Improved Modeling of Peptidic Foldamers Using a Quantum Chemical Parametrization Based on Torsional Minimum Energy Path Matching

**DOI:** 10.1002/cplu.201900180

**Published:** 2019-07-04

**Authors:** András Wacha, Tamás Beke‐Somfai, Tibor Nagy

**Affiliations:** ^1^ Institute of Materials and Environmental Chemistry Research Centre for Natural Sciences Hungarian Academy of Sciences H-1117 Budapest, Magyar tudósok körútja 2 Hungary

**Keywords:** β-peptides, foldamers, force field simulations, molecular dynamics, secondary structure

## Abstract

The increasing interest in novel foldamer constructs demands an accurate computational treatment on an extensive timescale. However, it is still a challenge to derive a force field (FF) that can reproduce the experimentally known fold while also allowing the spontaneous exploration of other structures. Here, aiming at a realistic reproduction of backbone torsional barriers, the relevant proper dihedrals of acyclic β^2^‐, β^3^‐ and β^2,3^‐amino acids were added to the CHARMM FF and optimized using a novel, self‐consistent iterative procedure based on quantum chemical relaxed scans. The new FF was validated by molecular dynamics simulations on three acyclic peptides. While they resided most of the time in their preferred fold (>80 % in helices and >50 % in hairpin), they also visited other conformations. Owing to the CHARMM36m‐consistent parametrization, the proposed extension is suitable for exploring new foldamer structures and assemblies, and their interactions with diverse biomolecules.

## Introduction

Foldamers experience a rapidly growing interest, where structures of increasing complexity provide potential for widespread applications^1^, ^2^ from nanotechnology^3^, ^4^, ^5^, ^6^ through biomedicine^7^, ^8^ to the development of drug molecules.^9^, ^10^, ^11^ By today, besides various secondary structures composed of single molecules,^12^ higher order oligomeric assemblies,^13^ some with protein‐like folds,^1^ have been constructed. However, design of scaffolds with increased level of complexity, requires molecular level insight to these constructs. In this respect, non‐natural peptidic molecules, foldamers, closely resembling natural peptides but also having important novel properties, are of high interest.^12^, ^14^, ^15^ β‐peptide foldamers, built from β‐amino acids, are the closest in homology to peptides composed of natural α‐amino acids and thus the most studied ones. Their structural diversity is similar to that of natural compounds, but they have significantly increased enzymatic stability, providing them great biomedical potential.^16^, ^17^

β‐peptide foldamers have a high propensity to form secondary structures, which are substantially different from those occurring in natural peptides, due to the presence of the additional methylene group in the backbone. Despite the numerous foldamer examples observed, efficient design of new structures and larger assemblies requires further molecular understanding. For natural peptides and proteins, there is an increasing amount of structural data assembled in e. g. the Protein Data Bank (PDB), which offers great reference for novel natural compounds. In contrast, in the case of foldameric systems even the commonly used CD‐ or IR‐spectra can be challenging to interpret,^15^, ^18^ especially in those cases where novel compounds or conformations are investigated. To support experiments, computational tools are regularly employed for the study of these systems, from quantum mechanical (QM) calculations via molecular mechanics (MM) to coarse‐grained descriptions of the energetics,^19^, ^20^ as well Molecular Dynamics (MD) and Monte Carlo (MC)^21^ The combination of MM and MD offers insight to structure and dynamics from the atomic level up to macroscopic assemblies on timescales up to microseconds.^18^, ^22^, ^23^, ^24^ Several well‐established MM force fields are available for biomacromolecules. Recent re‐parametrization of some force fields, for example the fine‐tuning of CMAP corrections in CHARMM36 m, makes them suitable to study also unstructured peptides.^25^, ^26^

Related, substantial progress has been made by several groups also in the development of FF parameters for β‐amino acids. van Gunsteren et al. combined MD with NMR results on various β‐peptide secondary structures.^18^, ^22^, ^24^, ^27^, ^28^ They extended the GROMOS 54A7 united atom force field for β‐peptides by adding chemically analogous atom types and adjusting the non‐bonded FF parameters responsible for intrachain hydrogen bonding.^29^, ^30^

For CHARMM22, Cui and coworkers optimized the barrier height parameters of the backbone torsion terms of β^3^‐amino acids using a multiobjective optimization method.^31^, ^32^, ^33^

Martinek et al. compared the behavior of mainly cyclic β‐peptides under the AMBER ff03 and the GAFF force fields using an explicit TIP3P water model.^34^ They used the program *antechamber* 35 to determine the required FF parameters and to assign partial charges to atoms based on the RESP^36^ method at the HF/6‐31G[AW2] (d) QM theoretical level.

However, the proper parametrization of β^2^‐ and β^2,3^‐amino acids has not been explicitly addressed so far, although this is important for progressing towards more advanced species in this area. Furthermore, as more refined non‐natural compounds are developed, recovering advanced structural and dynamic properties *in silico*, such as enabling conformational transitions for instance upon interaction with lipid bilayers, or upon binding to enzymes is becoming a key requirement. Higher‐order assemblies will also demand increasingly longer MD simulations, consequently the employed force field has to accurately reproduce the preferred conformation of flexible foldamer sequences. At the same time the FF also has to allow the exploration of the conformational space, rather than forcing sequences into one structure, as flexibility is a characteristic feature in the case of disordered sequences.

Based on these concepts, in the present work we extend the CHARMM36m FF with new backbone torsional parameters in order to make it capable of recovering the subtle and critical balance between different conformers of acyclic β‐peptide foldamers. An extension is developed in a rigorous and thorough manner starting from the *ab‐initio* torsional properties of the β‐peptide backbone built from simple β^2^‐, β^3^‐, β^2,3^‐amino acids and their achiral analogue residue, βA (or β‐hGly), rather than deriving it from experimental measurements on specific complex compounds. This theoretical approach, after proper validation against experimental data, is expected to better reconstruct and predict the dynamic folding behavior of foldamers containing β‐amino acids than FF parametrization based on the limited amount of experimental data.

The paper is structured as follows. In the first part, details are given on the FF development based on *ab initio* calculations. In the second part the proposed FF extension is validated and its good predictive ability is demonstrated by comparing simulation results to literature experimental data^24^, ^27^ obtained on three β‐peptide foldamers with known secondary structures. The performance of the new FF parameter set is also compared to that of the unmodified CHARMM36m FF and another extension, developed originally for CHARMM22 by Zhu et.al.^33^ It will be shown that the new FF extension is capable of recovering the dominant folds and other experimentally determined properties of three test foldamers while not hampering the peculiar folding/unfolding dynamics characteristic to these molecules.

## Methodology

Our aim is to extend and apply the well‐established CHARMM36m FF, which can accurately describe most important biomolecules: natural proteins and nucleic acids, sugars, lipids, and hydrocarbons. When coupled with the CGenFF force field, it covers most of the biologically relevant organic chemical space including small drug‐like molecules.^37^ Therefore, by deriving parameters only for the β‐amino acid backbone torsions, the extended FF should be readily applicable for the investigation of β‐peptidic foldamers even in exotic environments, such as phospholipid bilayers. The standard mathematical form of the potential function is the following [Eq. (1)]:^38^

(1) $$U\left(R\right)=\sum_{bonds}K_{b}{\left(b-b_{0}\right)}^{2}+\sum_{angles}K_{\theta }{\left(\theta -\theta_{0}\right)}^{2}+\sum_{Urey-Bradley}K_{S}{\left(S-S_{0}\right)}^{2}+\sum_{dihedrals}K_{\chi }^{prop.}\left(1+\cos\left(n_{\chi }-\chi_{0}\right)\right)+\sum_{impropers}K_{\chi }^{imp.}{\left(\chi -\chi_{0}\right)}^{2}+\sum_{nonbonded}\left\{\right\}\sum_{dihedrals}K_{\chi }^{prop.}\left(1+\cos\left(n_{\chi }-\chi_{0}\right)\right)+\sum_{impropers}K_{\chi }^{imp.}{\left(\chi -\chi_{0}\right)}^{2}+\sum_{nonbonded}\left\{\in_{ij}\left[\left(\frac{\sigma_{ij}}{r_{ij}}\right)-2{\left(\frac{\sigma_{ij}}{r_{ij}}\right)}^{6}\right]+\frac{q_{i}q_{j}}{4\pi \epsilon_{0}r_{ij}}\right\}$$

The potential energy of each dihedral term, defined by four, chemically bonded atoms is expressed with three parameters: the barrier half‐height $K_{\chi }$ , the multiplicity n and the zero phase $\chi_{0}$ . In conventional FFs, such as CHARMM, multiple proper dihedral terms with different multiplicities, varying between 1–6, can be provided for the same list of four atom types, similarly to a Fourier‐series expansion.

For naturally occurring α‐peptides and proteins, the CMAP correction in the CHARMM36m FF ensures a “perfect” fit of the *ab initio* energies of φ ‐ψ backbone torsions as it accounts for the finite flexibility of the force field using interpolation on a 2D grid. However, application of such a method for β‐peptides would require three‐dimensional maps, the determination of which would be overly expensive at the appropriate level of QM theory. Instead, we apply the standard method for determining FF parameters by fitting the QM potential energy of simple molecules with only proper dihedral terms at relevant points of the conformational space. We extend the CHARMM36m force field with new atom types and proper dihedral terms for the β‐peptide backbone while leaving its original parameters intact. It must be noted that the formation of intrachain hydrogen bonds is also an important factor in the folding process, however the additional methylene group in the backbone is not expected to induce significant changes that would require its re‐parametrization.

## Theoretical Considerations

### Backbone Torsions of Model β‐Peptides

**Figure 1:**
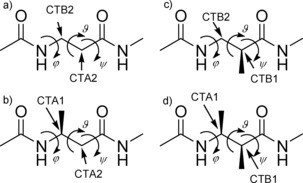
Torsion angles and atom types of the four diamides: (a) Ac‐βAla‐NHMe, (b) Ac‐(*S*)β^3^hAla‐NHMe, (c) Ac‐(*S*)β^2^hAla‐NHMe and (d) Ac‐(*2S*,*3S*)β^2,3^h(2Ala,3Ala)‐NHMe

As a comprehensive set of model compounds to be used in the parametrization, we chose four of the simplest acyclic protected β‐amino acids (shown in Figure 1): the achiral Ac‐βAla‐NHMe and three chiral ones with methyl side chains, Ac‐(*S*)β^3^hAla‐NHMe, Ac‐(*S*)β^2^hAla‐NHMe, and Ac‐(2*S*,3*S*)β^2,3^h(2Ala,3Ala)‐NHMe. These molecules with the investigated backbone torsion angles φ , ϑ and ψ are shown in the figure. In the following, we will briefly refer to them as β^0^, β^2^, β^3^ and β^2,3^ model peptides. We assume that the CHARMM36m FF describes the non‐bonded interaction of these smallest side‐chains the best, consequently it is expected that the refitted proper dihedral potentials along the backbone will be transferrable to longer side‐chains within the FF additivity approximation, while the differences in the torsional potential is intended to be captured by the CHARMM36m FF *via* the variations in the non‐bonded interactions of the side‐chains. Furthermore, note that although all chiral α‐ and β‐carbon atoms had an *S* absolute configuration, the results can be directly applied to amino acids with *R* chirality.

Backbone conformational space exploration of these molecules was done at the MP2/6‐31G(d) level in accordance with the development of the CHARMM36m force field. It is well‐known that high‐frequency stretching and bending vibrations adapt to the change of slow, large‐amplitude torsional motion, consequently the experienced barrier height is reduced during conformational dynamics and this can be the best modelled by relaxed scans of the backbone torsional angles. As a first step, the global minimum‐energy conformers were found, and then starting from them, 1D relaxed potential scans were carried out along each of the three torsion angles in 5° steps (i. e. 72 steps make a full turn). In order to explore “hysteresis” effects, two complete turns were done both clockwise and anticlockwise. Finally, the “global” torsional minimum energy paths (MEP) were extracted by selecting the lowest energy conformation for each value of the scanned angle, resulting in 4×3 MEPs, each consisting of 72 structures. Note, these MEPs, however, are not necessarily continuous, as they may contain nonadjacent geometries.

Compared to cyclic ones, simulation of acyclic β‐peptides requires more caution, as the higher backbone flexibility renders ordered secondary structures less stable. It is therefore reasonable to optimize those force field parameters – namely the backbone torsions involving β‐carbon atoms – which are exclusive for these compounds and are responsible for the emergence of various non‐standard secondary structures. Consequently, we have extended the CHARMM36(m) FF for β‐peptides composed of β^0^, β^2^, β^3^ and β^2,3^‐amino acids by introducing four new backbone carbon atom types. In the case of β^3^‐peptides, following the notation introduced earlier by Zhu et.al.,^33^ atom types CTA1 and CTA2 were used for the β and the α carbons (C_β_ and C_α_), respectively. These atom types are derived as copies of CT1 (aliphatic sp^3^ tertiary carbon) and CT2 (aliphatic sp^3^ secondary carbon) of the original FF. Similarly, CTB2 and CTB1 were created for the C_β_ and C_α_ atoms of the β^2^‐peptide. Based on analogous connectivities, these four atom types could also be used for the remaining two molecules as shown in Figure 1.

Atom types HB1 and HB2 were chosen for the hydrogen atoms attached to backbone carbons with (CTA1 and CTB1) or without (CTA2 and CTB2) side‐chains, respectively. Partial charges of the residues were assigned according to analogies with α‐peptides. The new parameters and topologies for all possible acyclic β‐amino acids with proteinogenic side‐chains are published separately, as detailed at the end of this paper.

### Dihedral Potential Terms

As stated above, the CHARMM‐type FF parametrization allows the use of more than one dihedral potential term with different multiplicities, strength parameters and offset phases for each torsion angle. Enumerating all torsions involving the newly proposed carbon atom types, altogether 12, 16–2=14, 16–2=14 and 21–8=13 dihedral angles can be identified in the β^0^, β^2^, β^3^, β^2,3^ model peptides, respectively, where the numbers are reduced by the number of torsions common with the preceding molecules in the list (e. g. H‐NH1‐CTB2‐HB2 in β^2^ is also in β^0^; see Table S3. in the Supporting Information).

If all six multiplicities were considered for each dihedral angle, it would correspond to $\left(12+14+14+13\right)\times 6=318$ dihedral terms and 318×2=636 optimizable parameters (all $K_{\chi }$ and $\chi_{0}$ ). Using increasingly more dihedral terms improves the agreement between the QM and MM energies at the fitted conformations. However, at some point overfitting occurs, which should be avoided as it introduces strong correlations between the parameters, induces large uncertainty of their fitted values and/or makes them unphysically large. Consequently, the corresponding large torsional forces will not compensate each other at other, not fitted conformations, which can result in an unphysical or unstable behavior at these geometries during MD simulations. Fortunately, the number of independent terms and parameters can be drastically reduced based on the following physical and chemical considerations.

1. Reversing the order of the four connected atoms in a torsion or mirroring the molecule through a plane, the calculated value of the corresponding dihedral angle changes sign. However, the proper dihedral potential energy must be independent of the order of the participating atoms (ijkl or lkji ), therefore the $\chi_{0}$ offset phase must also be insensitive to sign reversal. Consequently, it can only be either 0° or 180° (i. e. π ) [Eq. (2)]:

(2) $$\cos\left(n\left(\chi \right)\right)-\left\{0;\pi \right\}=\cos\left(n\left(-\chi \right)\right)+\left\{0;\pi \right\}=\cos\left(n\chi -\left\{0;\pi \right\}\right)$$

These choices, shown in curly brackets, also ensure that the energy of stereoisomeric amino acids and peptides that are mirror images of each other will be the same. Hence, the force field developed here, will be applicable to all stereoisomers.

2. Without loss of generality, the zero phase ($\chi_{0}$ ) can be arbitrarily set to 0, as switching between 0 and π is equivalent to changing the sign of the barrier height parameter as the following trigonometric identity holds [Eq. (3)]:

(3) $$\cos\left(n\chi -\left\{0;\pi \right\}\right)=-\cos\left(n\chi -\left(\pi ;0\right)\right).$$

At the end of the fitting procedure, the optimized value of those $K_{\chi }$ parameters that turned out to be negative, can be made positive by simultaneously switching $\chi_{0}$ from 0 to π . Note, this will shift the value of potential energy upward by a constant value of $-2K_{\chi }$ , which, however, has no effect on the dynamics [Eq. (4)]:

(4) $$K_{\chi }\left(1+\cos\left(n\chi -\left\{0;\pi \right\}\right)\right)-2K_{\chi }=-K_{\chi }\left(1+\cos\left(n\chi -\left\{\pi ;0\right\}\right)\right).$$

Together with the previous consideration, this leaves the $K_{\chi }$ barrier height as the only continuous, thus least‐squares fittable parameter.

3. Barrier height parameters of the backbone dihedral terms that involve aliphatic hydrogen atoms (atom types HB1 and HB2) are set to zero, as the position of such H atoms are primarily determined by the repulsion of geminal bonds (i. e. angle potentials) and much less affected by 1–4 neighbour (i. e. dihedral) interactions. Note, this simplification, which is also employed in the CHARMM force field, is applicable only to non‐methyl H atoms where the optimal value of the torsional angle is anchored by the non‐zero torsional (and non‐bonded) terms of the geminal non‐H atoms, whereas in the case of “terminal” HA3 atom types no such reference is present.

4. Due to the sp^2^ and sp^3^ hybridization states of the backbone atoms, only single, double, triple and sextuple multiplicities can be justified.

5. Due to the rigidity of the planar amide group, the torsion angles involving atom types around the same two central atoms are strongly correlated that induces strong correlations between the fitted values of the corresponding $K_{\chi }$ barrier height parameters, too. By assuming the planarity of the peptide bonds (amide groups) several torsion angles turn out to be supplementary pairs (i. e. making 180°). For such pairs of dihedral terms with the same odd multiplicities (n=2m+1 ) the same strength parameter and different starting phase (0°↔180°) should be used to make their contributions artificially constrained to each other [Eq. (5)]:

(5) $$K_{\chi }\left(1+\cos\left(\left(2m+1\right)\left(\pi -\chi \right)-\left\{0;\pi \right\}\right)\right)=K_{\chi }\left(1+\cos\left(\left(2m+1\right)\chi -\left\{\pi ;0\right\}\right)\right).$$

In the case of even multiplicities (n=2m ) the same barrier height parameter and starting phase should be used [Eq. (6)]:

(6) $$K_{\chi }\left(1+\cos\left(2m\left(\pi -\chi \right)-\left\{0;\pi \right\}\right)\right)=K{}_{\chi }\left(1+\cos\left(2m\chi -\left\{0;\pi \right\}\right)\right).$$

Such constraints are applied for instance in the case of the β^2^‐model peptide for the two torsions defined by atom types {C; H}‐NH1‐CTB2‐CTB1.

6. Due to the rigidity of the tetrahedral configuration around the backbone C_α_ and C_β_ atoms, the torsion angles that involve them as one or both of the central atoms, are not independent. The sp^3^ hybridization state of the α‐ and β‐carbons causes triplets of torsion angles with the same three common atoms to be separated by approximately 120° in most low‐energy geometries. This occurs in the β^2^‐model peptide for torsions NH1‐CTB2‐CTB1‐{C; CT3; HB1} of the χ=ϑ angles. Requiring once again their contributions to the potential energy to be the same [Eq. (7)]:

(7) $$K_{\chi }^{'}\left(1+\cos\left(n\left(\chi \pm \frac{2\pi }{\epsilon }\right)-\chi_{0}^{'}\right)\right)=K_{\chi }\left(1+\cos\left(n\chi -\chi_{0}\right)\right)+\mathrm{constant},$$

it turns out that the requirement that the initial phase must be 0 or π can only be met if n=3 or n=6 . In these cases, 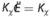 and 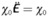 is found.

7. Finally, in order to choose the minimal set of multiplicities for each torsion, histograms were constructed for each of the torsion angles from the coordinate sets of all relaxed geometries yielded by the QM relaxed scans – i. e. not just those along the global MEPs (see Figures S6‐S9. in the Supporting Information). The required multiplicities for each torsion were determined considering that the most frequently occurring relaxed torsion angle values in the scans are necessarily around the minima of the torsional potential wells. For instance, it is found that the ϑ angles generally localize in close neighbourhoods of ±60°, ±90° and ±150°, which requires n=3 and 6 multiplicities.

Taking all the above considerations in account, 96 non‐zero barrier height parameters (including all multiplicities) remained, of which 38 were independent (see Tables 1‐4. for their lists and interdependences).

### Optimization Procedure

**Figure 2:**
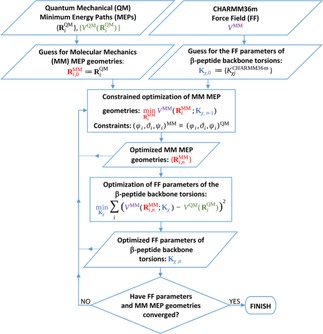
Self‐consistent optimization method employed for finding the optimal values for the torsional barrier height parameters. The optimized FF parameters (**K**_χ_) and MM MEP geometries ($R_{i}^{MM}$ ) at the beginning and at the end of the n ‐th iteration are denoted by subscript n-1 and n , respectively.

Once the multiplicities and the offset phases are chosen and the highly‐correlated dihedral terms are identified and bound together, the remaining independent barrier height parameters can be determined by fitting the MM torsional potentials to the QM energies along the MEPs. However, even if a good fit is achieved (e. g. within chemical accuracy: RMSD<1 kcal/mol) along the QM MEP, the real MM MEP can be significantly different from that of the QM one as conventional force fields miss some necessary coupling terms between stretching/bending and torsional motions. Consequently, instead of fitting the QM energies along the QM MEP geometries, it gives more realistic results if the QM MEP energies are matched with the MM MEP energies as a function of the dihedral angles. Such a fit provides the best possible MM reconstruction of the shape and height of the torsional barriers which are the key properties in determining the transition rates between various conformers. The optimal parameters were determined in the following iterative procedure (Figure 2):

1. The initial values of the proper dihedral potential parameters ($K_{\chi j}^{\;}$ ) were taken from those of analogous atom types, such as CT1 or CT2 in the CHARMM36m force field. All offset phases were chosen to be zero.
2. The initial set of starting geometries for the determination of the MM MEPs ($R_{i}^{MM}$ ) were taken from the QM MEPs ($R_{i}^{QM}$ ).
3. For the candidate set of force field parameters, the 12 MM MEPs along the 3–3 backbone dihedral angles of the 4 diamides were determined by constrained optimization using the steepest descent algorithm. While the QM MEPs were determined by fixing a single dihedral angle at 0, 5, … 360° values, the corresponding MM MEPs were determined by fixing all the three main backbone dihedrals (i. e. φ , ϑ and ψ ) simultaneously at their QM MEP values by applying 10^5^ kJ/mol/rad^2^ restraint potentials. The rest of the coordinates were left free to relax. This modification was necessary to prevent the MM MEPs from diverging far from the QM MEP during geometry optimization.
4. Using the candidate MM MEP geometries, an updated set of the barrier height parameters were determined by a least‐squares fit of the potential energy of the MM MEP to that of the QM MEP.
5. The geometries along the corresponding MM MEPs were stored as new starting conformations for the upcoming geometry optimizations with the updated barrier height parameters.
6. Steps 3–5 were repeated until the parameters and the energies converged. Convergence also meant that the MM parameters and MEPs had become self‐consistent.

## Results and Discussion

### Optimization Results

The optimized set of 38 independent barrier height parameters showed only few remaining strong correlations (see Figure S5. in the Supporting Information). The correlations between parameters corresponding to torsions around different pairs of backbone atoms (φ , ϑ or ψ ) were negligible. As the torsional potential is a linear function of the barrier height parameters, the remaining correlations greater than 0.9 or smaller than −0.9 have been removed by requiring the involved parameters to be equal or opposite of each other. However, as the sign of a barrier height parameter can be changed if its corresponding starting phase is flipped (0↔π ) according to point 2 in the above listed considerations for selecting proper dihedral potential terms, strongly anticorrelated parameters (i. e. r<-0.9 ) can be constrained to be the same (instead of opposite) by flipping the corresponding $\chi_{0}$ for one of them.

Tables 1–4 enumerate all of the torsions considered in the optimization process, along with their defining atom types and the chosen multiplicities. The optimized and sign‐adjusted half barrier heights, along with the final offset phases for all proper dihedral terms are also shown in the tables. Although no constraints were applied on the range of the barrier height parameters in the fitting procedure, all optimized values are well below 1 kcal/mol. This also confirms that the parameter correlations have been mostly eliminated. The last column of each table shows if the given half barrier height was an independent parameter in the fitting procedure (empty) or it had been constrained to be the same as another parameter before the fitting. Readily usable parameter and topology files for CHARMM and GROMACS are published separately, as detailed at the end of this paper.

**Table 1:** Fitted parameter values for the torsions of the achiral β‐diamide.

Torsion	Atom types				*n*	*K*_*χ*_ [kcal/mol]	*χ_0_* [°]	Constrained to
φ_1_^(0)^	C	NH1	CTB2	CTA2	1	0.27	180	–
					2	0.16	0	–
					3	0.29	180	–
φ_3_^(0)^	H	NH1	CTB2	CTA2	1	0.27	0	φ_1_^(0)^
					2	0.16	0	φ_1_^(0)^
					3	0.29	0	φ_1_^(0)^
ϑ_1_^(0)^	NH1	CTB2	CTA2	C	3	0.94	0	–
					6	0.07	0	–
ψ_1_^(0)^	CTB2	CTA2	C	NH1	1	0.68	0	–
					2	0.21	180	–
					3	0.13	180	–
ψ_2_^(0)^	CTB2	CTA2	C	O	1	0.68	180	ψ_1_^(0)^
					2	0.21	180	ψ_1_^(0)^
					3	0.13	0	ψ_1_^(0)^

**Table 2:** Fitted parameter values for the torsions of the β^2^‐diamide.

Torsion	Atom types				*n*	*K*_*χ*_ [kcal/mol]	*χ_0_* [°]	Constrained to
φ_1_^(2)^	C	NH1	CTB2	CTB1	1	0.11	0	–
					2	0.41	0	–
					3	0.42	180	–
					6	0.20	0	–
φ_3_^(2)^	H	NH1	CTB2	CTB1	1	0.11	180	φ_1_^(2)^
					2	0.41	0	φ_1_^(2)^
					3	0.42	0	φ_1_^(2)^
					6	0.20	0	φ_1_^(2)^
ϑ_1_^(2)^	NH1	CTB2	CTB1	C	3	0.43	0	–
					6	0.21	0	–
ϑ_2_^(2)^	NH1	CTB2	CTB1	CT3	3	0.43	0	ϑ_1_^(2)^
					6	0.21	0	ϑ_1_^(2)^
ψ_1_^(2)^	CTB2	CTB1	C	NH1	1	0.46	0	–
					2	0.26	0	–
					3	0.03	180	–
					6	0.01	0	–
ψ_2_^(2)^	CTB2	CTB1	C	O	1	0.46	180	ψ_1_^(2)^
					2	0.26	0	ψ_1_^(2)^
					3	0.03	0	ψ_1_^(2)^
					6	0.01	0	ψ_1_^(2)^
ψ_3_^(2)^	CT3	CTB1	C	NH1	1	0.51	0	–
					2	0.35	0	–
					3	0.03	180	ψ_1_^(2)^
					6	0.01	0	ψ_1_^(2)^
ψ_4_^(2)^	CT3	CTB1	C	O	1	0.51	180	ψ_3_^(2)^
					2	0.35	0	ψ_3_^(2)^
					3	0.03	0	ψ_1_^(2)^
					6	0.01	0	ψ_1_^(2)^

**Table 3:** Fitted parameter values for the torsions of the β^3^‐diamide.

Torsion	Atom types				n	Kχ (kcal/mol)	χ0 (°)	Constrained to
φ_1_^(3)^	C	NH1	CTA1	CTA2	1	0.18	180	–
					2	0.12	0	–
					3	0.03	0	–
					6	0.04	180	–
φ_2_^(3)^	C	NH1	CTA1	CT3	1	0.14	180	–
					2	0.04	0	–
					3	0.03	0	φ_1_^(3)^
					6	0.04	180	φ_1_^(3)^
φ_4_^(3)^	H	NH1	CTA1	CTA2	1	0.18	0	φ_1_^(3)^
					2	0.12	0	φ_1_^(3)^
					3	0.03	180	φ_1_^(3)^
					6	0.04	180	φ_1_^(3)^
φ_5_^(3)^	H	NH1	CTA1	CT3	1	0.14	0	φ_2_^(3)^
					2	0.04	0	φ_2_^(3)^
					3	0.03	180	φ_1_^(3)^
					6	0.04	180	φ_1_^(3)^
ϑ_1_^(3)^	NH1	CTA1	CTA2	C	3	0.54	0	–
					6	0.03	180	–
ϑ_3_^(3)^	CT3	CTA1	CTA2	C	3	0.54	0	ϑ_1_^(3)^
					6	0.03	180	ϑ_1_^(3)^
ϑ_5_^(3)^	HB1	CTA1	CTA2	C	3	0.54	0	ϑ_1_^(3)^
					6	0.03	180	ϑ_1_^(3)^
ψ_1_^(3)^	CTA1	CTA2	C	NH1	1	0.23	0	–
					2	0.36	180	–
					3	0.30	180	–
					6	0.02	0	–
ψ_2_^(3)^	CTA1	CTA2	C	O	1	0.23	180	ψ_1_^(3)^
					2	0.36	180	ψ_1_^(3)^
					3	0.30	0	ψ_1_^(3)^
					6	0.02	0	ψ_1_^(3)^

**Table 4:** Fitted parameter values for the torsions of the β^2,3^‐diamide.

Torsion	Atom types				n	Kχ [kcal/mol]	χ0 [°]	Constrained to
φ_1_^(2,3)^	C	NH1	CTA1	CTB1	1	0.35	180	–
					2	0.13	0	–
					3	0.03	0	φ_1_^(3)^
					6	0.04	180	φ_1_^(3)^
φ_4_^(2,3)^	H	NH1	CTA1	CTB1	1	0.35	0	φ_1_^(2,3)^
					2	0.13	0	φ_1_^(2,3)^
					3	0.03	180	φ_1_^(3)^
					6	0.04	180	φ_1_^(3)^
ϑ_1_^(2,3)^	NH1	CTA1	CTB1	C	3	0.24	0	–
					6	0.02	0	–
ϑ_2_^(2,3)^	NH1	CTA1	CTB1	CT3	3	0.24	0	ϑ_1_^(2,3)^
					6	0.02	0	ϑ_1_^(2,3)^
ϑ_4_^(2,3)^	CT3	CTA1	CTB1	C	3	0.24	0	ϑ_1_^(2,3)^
					6	0.02	0	ϑ_1_^(2,3)^
ϑ_5_^(2,3)^	CT3	CTA1	CTB1	CT3	3	0.24	0	ϑ_1_^(2,3)^
					6	0.02	0	ϑ_1_^(2,3)^
ψ_1_^(2,3)^	CTA1	CTB1	C	NH1	1	0.64	0	–
					2	0.06	180	–
					3	0.03	180	ψ_1_^(2)^
					6	0.01	0	ψ_1_^(2)^
ψ_2_^(2,3)^	CTA1	CTB1	C	O	1	0.64	180	ψ_1_^(2,3)^
					2	0.06	180	ψ_1_^(2,3)^
					3	0.03	0	ψ_1_^(2)^
					6	0.01	0	ψ_1_^(2)^

**Figure 3:**
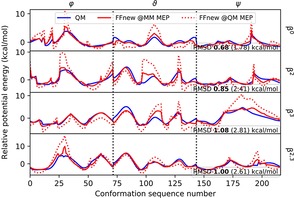
Fit of the QM (solid blue line) and MM (red lines) potential energies of the Ac‐βAla‐NHMe (“β^0^”), Ac‐(*S*)β^2^hAla‐NHMe (“β^2^”), Ac‐(*S*)β^3^hAla‐NHMe (“β^3^”) and Ac‐(2*S*,3*S*)β^2,3^‐h(2Ala,3Ala)‐NHMe (“β^2,3^”) peptides (enumerated from top to bottom). The MM potential energies were calculated on the MM MEP (solid red line, RMSD in boldface) and the QM MEP (dotted red line, RMSD in regular text).

The quality of the fit between the QM and MM potential energies is shown in Figure 3. A close matching with 0.914 kcal/mol overall RMS difference of the two curves is seen from the figure in the case of most elements of the MM MEPs. Though at some instances there are slight discrepancies in the height of the barriers between two neighboring torsional minima, the positions of the minima and maxima are nearly the same, and local thermal fluctuations should still enable the system to cross over between two neighboring states.

### Validation

In order to test the validity and applicability of the newly developed parameter set for β‐peptide torsions, physical quantities calculated from simulated trajectories are compared to experimental data and simulation results available in the literature. The performance of our FF extension is assessed in a broader sense, by comparing the results obtained by using it (denoted in the following by **FFnew**) with those obtained by employing two other parametrizations, namely the original CHARMM36m force field (**FFc36**) and the extension developed by Zhu et.al. (**FFZhu**).^33^ For an unbiased comparison, in all the three cases, the same partial charges for all atoms and the same atom types for the rest of the atoms (not C_α_ or C_β_) were taken. These were assigned by using analogies with α‐amino acid residues found in the residue topology database of the CHARMM force field (more information on the derivation of β‐residue topologies for all possible acyclic β‐amino acids with proteinogenic sidechains are included in the Supporting Information).

#### 14‐Helical Secondary Structure of the “VALXVAL” Heptapeptide

**Figure 4:**
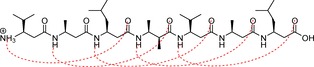
The chemical structure of the (*S*)β^3^hVal‐(*S*)β^3^hAla‐(*S*)β^3^hLeu‐(2*S*,3*S*)β^2,3^h(2Ala,3Ala)‐(*S*)β^3^hVal‐(*S*)β^3^hAla‐(*S*)β^3^hLeu heptapeptide (denoted as “VALXVAL”), which prefers forming a helix with 14‐membered hydrogen bonded pseudorings (illustrated with dashed lines).

To assess the performance and compare the reliability of the chosen three sets of parameters, the “VALXVAL” heptapeptide (Figure 4), which has been used extensively in experimental and also in MD studies,^29^, ^30^, ^33^, ^39^ was investigated. The dominant fold of this molecule in methanol was found by NMR experiments to be a 14‐helix (abbreviated H14, corresponding to an i→i+2 intrachain hydrogen bond pattern, illustrated in the figure with dashed lines).^40^

MD simulations for FF validation were preceded by a careful preparation procedure, executed separately for all the three force fields. The peptide, initially built in a fully extended conformation, had been manually folded into its H14 state by adjusting the φ , ϑ , ψ backbone torsion angles to values published by Beke et.al.,^41^ which are in good agreement with experimental values.^42^, ^43^ Particulars on the preparatory steps and the equilibration procedure can be found in the Supporting Information. Production MD runs were carried out in the NPT ensemble at 300 K temperature and 1 bar pressure, with 2 fs timestep. In order to allow the heptapeptide to explore the available conformational space, and to test the stability of its fold, no artificial geometric restraints were applied in the production runs. For assessing reproducibility, five independent simulations have been performed, in total nearly 2 μs for each force field.

**Figure 5:**
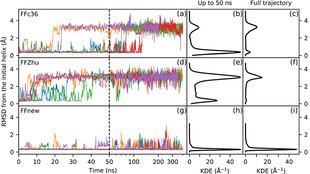
Time evolution (left panel) and Gaussian kernel density estimation (KDE, central and right panels) of the RMS deviation of the backbone atom positions of the “VALXVAL” heptapeptide from its initial H14 helix structure in residues 2–6 for three different parametrizations of the backbone torsions: original CHARMM36m (FFc36; a, b, c), Zhu et. al. (FFZhu; d, e, f), this work (FFnew; g, h, i). The results for the five trajectories are plotted with different colors. The KDEs in the central plots (b, e, h) were calculated from only the first 50∼ns, while on the right (c, f, i) from the full trajectory. A 0.76 Å KDE bandwidth has been chosen using Scott's rule.

The time evolution of the peptide under simulation is visualized in Figure 5. The left panel of the figure shows the root‐mean‐square deviation of the backbone atom positions of the core 2–6 residues from the initial helical conformation. In the first 30 ns all three force fields predict the same, expected behavior, more‐or‐less keeping the helical fold. Later on, however, the simulations with FFc36 and FFZhu show complete unfolding, i. e. the structure never folds back permanently to H14 throughout the simulation time. In contrast, the five parallel simulations with FFnew show that the helical conformation stays stable, in close agreement with the experimental results.

**Figure 6:**
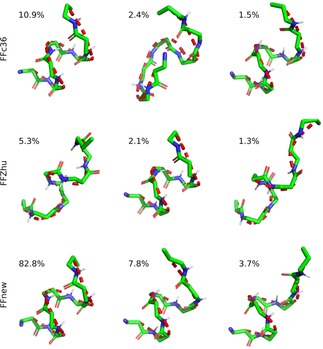
Central representative elements of the three most populated clusters and the respective occupancy ratios of the “VALXVAL” peptide, simulated at 300 K, 1 bar for nearly 2 μs.

Conformational clustering allows a more in‐depth study on the folding‐unfolding equilibrium. To this end, the five parallel trajectories were concatenated, and the cluster analysis was performed on the combined histories. Frames taken at every 0.1 ns were sorted into clusters based on the RMS distance of the backbone atoms in residues 2–6, using the algorithm proposed by Daura et.al.,^28^ with 1 Å cutoff distance. The central representative elements, i. e. those which lie in the middle in terms of RMS distance, of the three most populated clusters are shown in Figure 6. Under FFnew, the fully‐ordered H14 structure is remarkably stable, accounting for 82.8 % of the full trajectory. Alongside this, there is also a significant contribution from less‐ordered helices in the next 2 most populated clusters. In comparison, the most populated clusters obtained with the other two force fields are somewhat looser variants of the H14 helix. Additionally, their contributions are comparatively low, 10.9 % and 5.3 %, for the FFc36 and FFZhu, respectively.

It has also been found that in order to cover 95 % of the combined trajectories, just 3 clusters are needed in the case of our force field extension, while this number is much larger, 4296 for FFc36 and 5904 for FFZhu.

Assuming that the system has no intermediate states between the folded and unfolded ones, the Gibbs free energy of folding for the peptide can be estimated from the trajectory as $\Delta G_{\mathrm{folding}}=-k_{b}Tln\left(p_{\mathrm{folded}}/\left(1-p_{\mathrm{folded}}\right)\right)$ ,

where $p_{\mathrm{folded}}$ is the relative probability of the folded state, approximated by the fraction of the trajectory where the backbone atom RMS distance from the initial helical configuration is less than 1 Å. Using our parameters, the obtained value is −1.91 kcal/mol, clearly showing the thermodynamic stability of the helical conformation. This result can be compared to those obtained by Huang et.al.^29^ on the same heptapeptide, at 340 K temperature, with three different versions of the GROMOS united atom FF. Using the same RMSD threshold to separate folded and unfolded conformations, they have obtained −0.36, −0.88 and −1.29 kcal/mol with the 45A3, 53A6 and 54A7 versions of the FF, respectively. 1The choice of the threshold is somewhat arbitrary, though. For the dependence of the Gibbs free energy of folding on the RMSD threshold, please refer to the Supporting Information.


**Figure 7:**
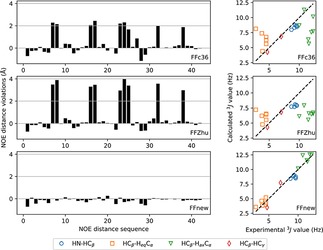
NOE distance violations (left) and ${}^{3}J$ coupling constants (right) of the heptapeptide according to the original CHARMM36m force field (FFc36, top), the parametrization proposed by Zhu et.al. (FFZhu, center) and this work (FFnew, bottom). All available frames of the trajectories were used for the averaging.

We have also investigated how consistent are the obtained trajectories with the published experimental NMR results on this peptide.^27^ NOE distances and ${}^{3}J$ couplings have been calculated from the trajectories. NOE distance violations were calculated according to ${\left\langle r^{-6}\right\rangle }^{-1/6}-r_{0}$ , where r is the instantaneous interproton distance and $r_{0}$ is the experimental NOE distance reported by Daura et.al..^27^ The violations are shown graphically in Figure 7 (and numerically in the Supporting Information). The ${}^{3}J$ coupling constants were derived from the corresponding torsion angles using the Karplus relation:^44^

$${}^{3}J\left(H,H\right)=\left\langle a\;{cos}^{2}\theta +b\;cos\theta +c\right\rangle ,$$

where a =6.4 Hz, b =−1.4 Hz and c =1.9 Hz for the calculation of ${}^{3}J\left(HN,HC\right)$ and a =9.5 Hz, b =−1.6 Hz and c =1.8 Hz for ${}^{3}J\left(HC,HC\right)$ .^45^, ^46^, ^47^ The averaging is done on all frames of the trajectories.

**Figure 8:**
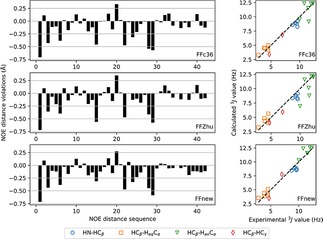
NOE distance violations (left) and ${}^{3}J$ coupling constants (right) of the heptapeptide according to the original CHARMM36m force field (FFc36, top), the parametrization proposed by Zhu et.al. (FFZhu, center) and this work (FFnew, bottom). Only the “folded” part of the trajectories (backbone atom position RMSD less than 1 Å from the initial helical configuration) were considered in the averaging.

Due to the upper bound nature of NOE distances, only positive violations are relevant. The sum of the positive NOE violations obtained by using the new, extended force field is less than one Ångström, 0.84 Å, significantly better than the values 22.5 and 34.1 Å obtained using FFc36 and FFZhu, respectively, due to the unfolding effect seen above. If the averaging is limited to the “folded” state (backbone atom position RMSD less than 1 Å from the initial, helical conformation, Figure 8), FFnew still performs better than the other two FFs (0.11 Å against 0.12 and 0.13 Å, respectively).

Looking only at the “folded” state, the largest positive NOE violation is found with all three parameter sets for the distance between the equatorial proton of the α‐carbon in residue 3 and the amide hydrogen of the central, β^2,3^ residue (see the table in the Supporting Information). Using FFnew, this is reduced from 0.33 Å to 0.27 Å.

In terms of the ${}^{3}J$ ‐coupling constants (right panels in Figure 7), FFnew also performs much better, with 0.56 Hz average absolute deviation, than FFc36 and FFZhu, where the mean absolute deviations are 2.11 and 2.69 Hz, respectively.

The presence or absence of intramolecular hydrogen bonds have also been determined from the trajectories. A donor and an acceptor atom were considered H‐bonded when the donor‐hydrogen‐acceptor angle was greater than 125° and the hydrogen‐acceptor distance less than 2.4 Å. Table 5. shows the proportions of the frames in which the various H‐bonds were present. According to the results, FFnew reports greater populations for H‐bonds found experimentally and lower values for nonexistent ones, than either FFc36 or FFZhu, which show values contradicting the experimental observations. The only exception from this is the hydrogen bond nearest to the C‐terminus. Interestingly, the simulation in all three cases prefers the H‐bond between the N atom of the 5th residue and the C‐terminal carbonyl O (NH(5)‐O1(7)), over an H‐bond with the protonated O (NH(5)‐O2(7)). The same behavior was found by Daura et.al.^27^ using the GROMOS force field.

**Table 5:** Intramolecular hydrogen bond populations.

Index	Donor	Acceptor	H‐bond populations [%]			
			NMR	FFc36	FFZhu	FFnew
1	NH(1)	O(2)	+	3.77	1.28	4.16
2	NH(1)	O(3)	+	12.67	4.87	70.34
3	NH(2)	O(4)	+	14.57	4.43	96.99
4	NH(3)	O(5)	+	14.28	4.28	95.34
5	NH(4)	O(5)	–	0.45	1.33	0.57
6	NH(4)	O(6)	+	11.69	2.30	83.52
7	NH(5)	O1(7)	–	4.41	0.84	59.42
8	NH(5)	O2(7)	+	0.10	0.04	0.70
9	NH(7)	O(5)	–	14.04	14.07	0.00

The pattern of the intrachain hydrogen bonds preserving the helical structure is characteristic to the type of the secondary structure. Table 6 classifies the full trajectories according to the number of i→i+2 hydrogen bonds of the 14‐helix. In this respect, too, the new FF parametrization outperforms the other two, as under those, the peptide spends about 90 % of the time in a completely unfolded state, without any stabilizing hydrogen bonds. On the contrary, in the case of FFnew the heptapeptide spends nearly 95 % of the simulation time in states where 3, 4 or even 5 H‐bonds stabilize the structure, and accordingly the ratios of populations with 2 : 3 : 4 : 5 H‐bonds (not shown here) have converged to the same values regardless of the starting conformation.

**Table 6:** Populations of the hydrogen bonds characteristic for the 14‐helix from simulations with the three kinds of FF parametrizations and two starting conformations.

Number of *i→i+2* H‐bonds	Populations with H‐bonds (%)					
	FFc36		FFZhu		FFnew	
	from H14	from linear.	from H14	from linear	from H14	from linear
0	82.27	95.13	92.70	95.14	0.14	17.78
1	2.53	1.66	2.91	3.09	0.82	1.15
2	1.85	0.26	1.43	0.61	5.61	4.43
3	4.52	1.00	1.34	0.65	18.70	15.16
4	6.18	2.10	1.14	0.44	35.46	29.83
5	2.64	2.45	0.48	0.07	39.26	31.63

**Figure 9:**
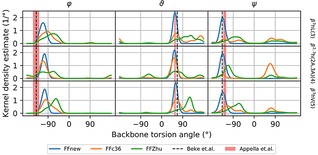
Backbone torsion angle distributions of the three central residues of the “VALXVAL” peptide. Estimations for the distribution densities are given using a von Mises (cyclic Gaussian) kernel with 5° band width.

The helical structure is also characterized by the torsion angles of the peptide backbones, in a similar fashion as the Ramachandran map of natural peptides and proteins. The characteristic backbone torsion angles of the H14 have been reported to be −140.3°, 66.5° and −136.8° and −141(13)°, 57(3)° and −126(6)° by Beke et.al.^41^ from theoretical computations and by Appella et.al.^43^ based on X‐ray diffraction experiments on crystallized samples, respectively. Figure 9 shows these values graphically together with the distributions of the three angles calculated from our MD trajectories for the three central residues, where chain end effects are negligible. For an extended comparison considering all residues, see Figure S10. in the Supporting Information.

The distribution of the ϑ and ψ angles match the published experimental/theoretical data satisfactorily for FFnew, while the other two FFs show nonzero torsion angle populations far from the expected values. The first dihedral angle (φ , centered on the N and the C_β_ atoms), however, is slightly overestimated by all three force fields, but FFnew seems to be the closest to the expectations. The discrepancy might be ascribed to methodological differences: the *ab initio* study of Beke et. al. has been dealing with lowest‐energy structures *in vacuo*, while Appella et.al. have obtained the torsion angles from X‐ray diffraction results on oligomers of cyclic β‐amino acids (trans‐2‐aminocyclohexanecarboxylic acid, ACHC), where both the crystallization and the conformational restraints of the cyclohexane ring can influence the actual dihedral angles.

The surprising stability of secondary structures shown by β‐peptides is well‐known.^16^, ^48^, ^49^, ^50^, ^51^ The above results induced us to test if the H14 conformation can evolve from an unfolded state. We have performed the same simulations on the β‐heptapeptide as before, but starting in a fully extended conformation. For all three FFs, five independent runs were done, 1 μs each, once again without applying any artificial constraints on the folding. The reason for the longer simulation time (1 μs vs 400 ns) was to allow the observation of folding for all FFs).

**Figure 10:**
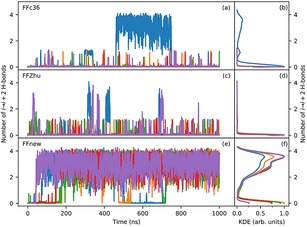
Time‐evolution (left panels) and normalized Gaussian kernel density estimation (KDE, right panels) of the number of backbone hydrogen bonds corresponding to the H14 fold of the “VALXVAL” peptide under the three force‐fields (FFc36: a,b; FFZhu: c,d; FFnew: e,f). The simulations have been started from a fully extended configuration. A 0.115 bandwidth was chosen for KDE using Scott's rule. For clarity, the KDEs have been divided by their maximum value.

Figure 10 shows the number of i→i+2 hydrogen bonds, calculated with the switching function approach which is also implemented in PLUMED (using $r_{0}$ =2 Å, n =6, m =10).^52^ All of the five independent runs performed with FFnew show that the hydrogen bonds characteristic for the H14 fold appear after 200 ns in the worst case. Some runs even produce an unfolding‐refolding behavior throughout the simulation. The hydrogen bond network is absent in the case of the other two parametrizations, only one run performed with FFc36 shows a significant period where the i→i+2 H‐bonds are present. The i→i+2 H‐bond populations are shown alongside the results from the previous simulations in Table 6. The percentages of FFc36 and FFZhu are similar regardless of the starting conformation, due to the quick unfolding and the lack of folding in the helical and the extended case, respectively. In the case of FFnew the values are also similar to the results obtained when started from a helical conformation. The unfolded state has a much larger percentage, due to the bias introduced by the choice of the initial state for finite simulation times. Accordingly, the values for the folded states are a bit smaller. The similarity of the H‐bond statistics illustrates that the sampling is independent from the starting conformation for all three FF variants.

**Figure 11:**
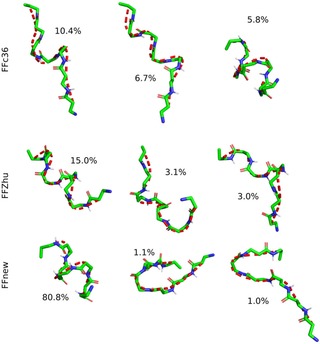
Central representative elements of the three most populated clusters and the respective occupancy ratios of the “VALXVAL” peptide, simulated at 300 K, 1 bar for 5 μs. The simulation has been started from a fully extended conformation.

The results of conformational clustering are shown in Figure 11. In contrast to the simulation started from the helical conformation, only the third most populated cluster is a helix with FFc36. FFZhu has a somewhat looser helical structure as its most probable conformation (15 % of the simulation time), whereas FFnew produces the expected helix in 80 % of the time.

**Figure 12:**
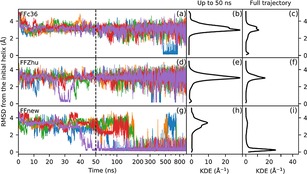
Time evolution (left panel) and distribution (right panel) of the RMS deviation of the backbone atom positions from the H14 structure in residues 2–5 of the VALXVAL heptapeptide at 300 K and 1 bar for three different sets of torsion parameters: original CHARMM36m (FFc36; a, b), parameters by Zhu et. al. (FFZhu; c, d) and this work (FFnew; e, f). Simulations were started from an extended conformation. The results for the five trajectories are plotted with different colors. A 1.1 Å KDE bandwidth has been chosen using Scott's rule.

The RMS deviation of the backbone atoms of residues 2–6 from the helical structure in the most populated cluster of FFnew is shown in Figure 12. Although the reference structure is not an experimentally validated conformation, the curves illustrate well the helicity of the conformation of the peptide.

We see the same behavior as with the number of hydrogen bonds, namely that with FFnew the peptide folds after a short time into the H14 conformation and remains mostly in the neighborhood of this structure, with sporadic unfolding/refolding in two of the five runs. With the other two FF variants the heptapeptide does not fold: the helical state can only observed infrequently, and even then only for short times.

The same conformance in terms of the experimentally obtainable quantities (NOE violations and ${}^{3}J$ ‐couplings) has been found for FFnew, while the other two FFs performed in a similar way as when the simulations were started from the helical state (see Supporting information).

#### β‐hexapeptide with Disubstituted Residues Adopting a Hairpin Structure

**Figure 13:**
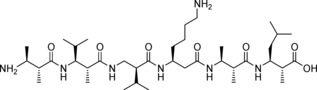
The structure of the (2*R*,3*S*)β^2,3^h(2Ala,3Ala)‐(2*R*,3*S*)β^2,3^h(2Ala,3Val)‐(*S*)β^2^hVal‐(*S*)β^3^hLys‐(2*R*,3*S*)β^2,3^h(2Ala,3Ala)‐(2*R*,3*S*)β^2,3^h(2Ala,3Leu) β‐hexapeptide (“HP6”), which prefers a hairpin structure.

Seebach and his coworkers noticed that β‐peptidic sequences consisting entirely of β^2,3^ residues where the chirality of the α and β carbons are opposite (which they termed as *‘unlike’* β‐amino acid residues) aggregate into pleated sheets, which are held together by interchain hydrogen bonding, as they prefer elongated conformations over helices.^53^ If a turn is induced by inserting an (*S*)‐β^2^‐(*S*)‐β^3^ dipeptide into the sequence, such as shown in Figure 13, the peptide (referred to in the following as **HP6**), folds into a hairpin structure.

HP6 was extensively studied as a test example for various versions of the GROMOS force field and the simulation results were compared to NMR measurements.^24^, ^29^, ^39^

After similar preparations as done on the VALXVAL heptapeptide above, we performed MD simulations on HP6 with all three force fields at 300 K temperature and 1 bar pressure (for details, see the Supplementary Information).

**Figure 14:**
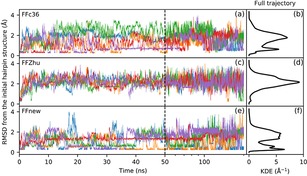
Time evolution (left panel) and Gaussian kernel density estimation (right panel) of the RMS deviation of the backbone atom positions from the initial hairpin structure in residues 2–5 of the HP6 hexapeptide at 300 K and 1 bar for three different sets of torsion parameters: original CHARMM36m (FFc36; a, b), parameters by Zhu et. al. (FFZhu; c, d) and this work (FFnew; e, f). The results for the five trajectories are plotted with different colors. A 0.6 Å KDE bandwidth has been chosen using Scott's rule.

Figure 14 presents the unfolding dynamics of the hairpin through the RMS deviation of the backbone atom positions from the initial hairpin structure in the four central residues. Similarly to VALXVAL, the FFc36 parameter set results in an irreversible unfolding also for HP6 after the first 50 ns. Using FFZhu, the peptide unfolds almost instantaneously, and never returns to a structure resembling the initial, hairpin conformation. The highest peak of the population density plot is at 0.26 Å RMSD with FFnew, meaning that with this parameter set the trajectories sample the hairpin structure with higher probability than with the others (peak positions: FFZhu: 2.1 Å, FFc36: 1.8 Å and 0.63 Å).

**Figure 15:**
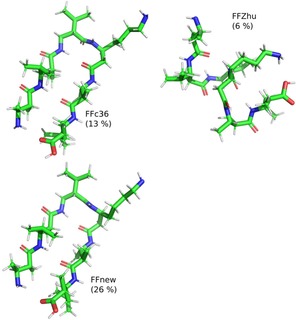
Central representative elements of the most populated clusters from the combined trajectories. The relative population of the clusters is shown in parentheses.

Again, the five distinct trajectories were combined and cluster analysis was carried out applying the same method as used for the helical heptapeptide.^28^ The central representative elements of the most populated clusters are shown in Figure 15, along with their relative population. Using FFnew, the peptide resides in the first cluster in 26 % of the total sampling time, which is twice as long as when FFc36 is used. Both cases give hairpins as the most probable conformations. This contrasts with what we have found with FFZhu, where the most probable conformation is some kind of helix. Additionally, it was found that with FFnew, 95 % of the combined trajectory is covered by 74 clusters, which in comparison to the helical heptapeptide shows that for this peptide the folded conformation is less preferred. The irreversible unfolding with the other two FFs results in greater molecular flexibility, therefore much larger number of clusters for covering 95 % of the trajectory (203 and 629 for the FFc36 and FFZhu, respectively).

The Gibbs free energy of folding is obtained from the trajectories using a 0.8 Å RMSD threshold, as proposed by Huang et.al.^29^ The value 0.56 kcal/mol, predicted by FFnew, is lower than what is found with FFc36 (0.92 kcal/mol) and much lower than with FFZhu (2.50 kcal/mol), confirming once again the relative stability of the folded state with the new FF parameter set. In comparison, Huang et.al.^29^ reported 2.0, 1.4 and 1.0 kcal/mol with GROMOS 45A3, 53A6 and 54A7 at 340 K, respectively.

**Figure 16:**
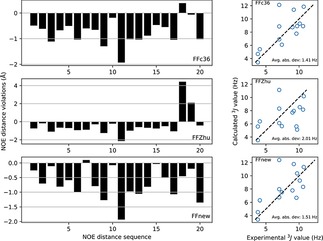
NOE distance violations and ${}^{3}J$ couplings of the hexapeptide hairpin according to the original CHARMM36m force field parameters (top), and the torsion parameters derived by Zhu et.al. (center) and this work (bottom). All available frames of the trajectories have been used in the averaging.

Comparison of the simulations to NMR experiments in terms of NOE distance violations and ${}^{3}J$ couplings was carried out for this peptide as well. In Figure 16, it is shown that FFnew performs slightly better than FFc36, and much better than FFZhu. The largest positive NOE violation is found for the distance between protons on the α‐carbon of the first residue and the β‐carbon of the last residue (0.38 Å, see the Supporting Information for all NOE violations). This is eliminated with the newly developed parameter set, where the largest positive NOE distance violation is between the amide proton of residue 3 and the β‐proton of residue 2 (0.10 Å). In terms of ${}^{3}J$ couplings, all three force fields perform similarly, with 1.41, 2.01 and 1.51 Hz average absolute deviation found by FFc36, FFZhu and FFnew, respectively.

Because the hairpin hexapeptide prefers the folded state less than the helical heptapeptide, the same stability of the dominant fold cannot be expected. A good measure of the stability is the population of the hydrogen bonds responsible for the hairpin conformation, listed in Table 7. It is found that the innermost hydrogen bond (between residues 3 and 4, part of a 10‐membered loop) is populated during nearly half of the sampling time for FFnew, while the other two FFs report much less statistical weight. Using the proposed FF extension, the occupancies of this and the other two H‐bonds are also significantly larger, hinting that the dominant fold is better recovered in our case.

**Table 7:** Populations of the hydrogen bonds defining the hairpin conformation of the hexapeptide.

Donor	Acceptor	H‐bond populations [%]		
		FFc36	FFZhu	FFnew
NH(3)	O(4)	43.42	13.66	51.98
NH(2)	O(5)	13.86	1.10	22.77
NH(1)	O(6)	1.40	0.16	2.85

#### The Helical Conformation of a Water‐Soluble β‐Undecapeptide

**Figure 17:**
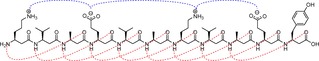
The structure of the amphiphilic (*S*)β^3^hOrn‐(*S*)β^3^hVal‐(*S*)β^3^hAla‐(*S*)β^3^hGlu‐(*S*)β^3^hVal‐(*S*)β^3^hAla‐(*S*)β^3^hOrn‐(*S*)β^3^hVal‐(*S*)β^3^hAla‐(*S*)β^3^hGlu‐(*S*)β^3^hTyr β‐undecapeptide (“AH”), adopting a helical structure in aqueous milieu. Red dashed lines show the *i*→*i*+2 H‐bonding network, blue dashed lines the salt bridges formed in the H14 helix.

Research on β‐peptides was traditionally focusing on hydrophobic side‐chains. Application of these molecules as antimicrobial or anticancer drugs, however, often requires water‐soluble molecules. Recently, several hydrophilic/amphiphilic β‐peptides were reported to fold into stable helical structures.^1^, ^54^, ^55^, ^56^ Aiming at a more complete and representative validation of our force field, we have chosen one of the water‐soluble β‐undecapeptides, which forms a H14 helix in water (denoted later on as “AH”, schematically represented in Figure 17).^55^ The amino acid sequence of this peptide has been designed in a way that in the H14 helix, where three amino acids correspond to a full turn, the hydrophilic side‐chains can form salt bridges, which in turn stabilize the fold.

**Figure 18:**
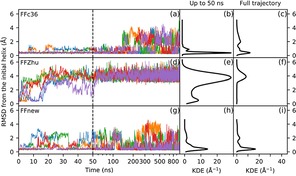
Time evolution (left panel) and distribution (center and right panels) of the RMS deviation of the backbone atom positions from the H14 structure in residues 2–10 of the AH undecapeptide at 300 K and 1 bar for three different sets of torsion parameters: original CHARMM36m (FFc36; a, b), parameters by Zhu et. al. (FFZhu; c, d) and this work (FFnew; e, f). Simulations were started from an extended conformation. A 0.115 bandwidth was chosen for KDE using Scott's rule. The results for the five trajectories are plotted with different colors.

The same simulation has been carried out as for the “VALXVAL” peptide, started from the helical state, only water has been used for solvation instead of methanol. The RMS deviations of the backbone atoms of residues 2–10 from the initial, helical conformation throughout the runs is shown in Figure 18.

As seen from the figure, the expected secondary structure is retained under FFnew. However, in contrast to what was observed for “VALXVAL”, FFc36 also keeps the helical conformation, possibly because of the role of side‐chains in the stability. The peptide quickly unfolds if FFZhu is used, however, maybe due to the incompatibilities between CHARMM22 and CHARMM36m.

**Figure 19:**
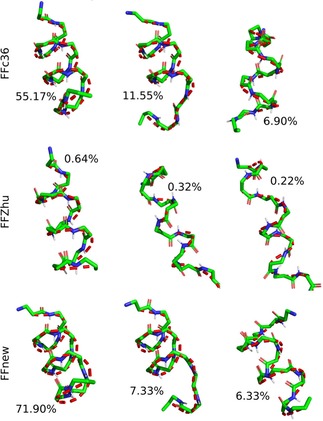
Central representative elements of the three most populated clusters and the respective occupancy ratios of the “AH” peptide, simulated at 300 K, 1 bar for 3×5×1 μs.

The three most populated conformational clusters are shown in Figure 19. for each FF variant. As in the previous cases, only the backbone atoms of the inner residues (2–10 in this case) have been considered. Both FFc36 and FFnew have helices as their most probable conformation with a decent population – 55 and 72 %, respectively.

The populations of the i→i+2 H‐bonds characteristic for the H14 fold are shown in Table 8. In this respect, FFnew reproduces the folded state ($N_{i\to i+1}>6)$ with a slightly larger statistical weight than FFc36.

**Table 8:** Populations of the hydrogen bonds characteristic for the 14‐helix from simulations with the three kinds of FF parametrizations.

Number of *i→i+2* H‐bonds	Populations with H‐bonds [%]		
	FFc36	FFZhu	FFnew
0	0.60	96.28	0.01
1	0.29	1.01	0.08
2	2.11	0.61	0.69
3	6.17	0.46	3.21
4	11.05	0.56	8.07
5	19.73	0.41	16.14
6	17.69	0.30	19.67
7	20.02	0.26	24.19
8	20.70	0.10	25.35
9	1.64	0.02	2.62

## Conclusion

We have developed a new extension to the CHARMM36m force field for achiral, as well as β^2^‐, β^3^‐ and β^2,3^‐substituted acyclic β‐amino acids and peptides constructed from these. The barrier height parameters of the backbone torsions have been re‐parametrized against geometries and potential energies obtained from relaxed MP2/6‐31G(d) scans of the three torsion angles. Special focus was put on the close reproduction of the QM barrier heights between neighboring torsional states, in order to allow realistic exploration of the conformational space and sampling of the experimentally determined dominant folds.

The parameter set has been validated against experimental results on a β‐heptapeptide, and it was found that the dominant 14‐helix determined from NMR measurements is reproduced in the largest part of the MD trajectories of nearly 2 μs total simulation time. This is in contrast with the original, unmodified CHARMM36m force field and another, previously published similar extension for β‐peptides, where the simulations resulted in the complete, irreversible unfolding of the peptide after the first 40 ns. Experimentally measurable quantities such as NOE distances and ${}^{3}J$ couplings have been determined from the trajectories and compared to the already published results in the literature. Our proposed force field extension clearly outperformed the other two FFs, due to the irreversible unfolding found in the latter. Even if the trajectories are limited only to initial simulation periods, where the folded state is still present, our proposed extension still performs better than the other two variants. When the same simulation is repeated from a fully extended initial conformation, the β‐heptapeptide folds into its preferred H14 conformation under the new FF, while failing to fold when the other two parametrizations are used, even over longer simulation times of multiple runs (5 times 1 μs).

A β‐hexapeptide, containing β^2^, β^3^ and β^2,3^‐residues and which has been reported to assume a hairpin conformation, has also been analyzed with the new FF extension. Again, in contrast with the irreversible unfolding found with other two FFs, the peptide, known to be much more flexible regarding to its secondary structure, spends much of its time near the dominant hairpin conformation while exploring other regions of the conformational space. The new force field performs better than its ancestors in terms of NMR quantities (NOE distances and ${}^{3}J$ couplings) in this case, too. While the performance of the unmodified CHARMM36m FF was only slightly worse than our proposed extension in this sense, though still failed to reproduce the dominant hairpin fold, the extension published by Zhu et.al.,^33^ originally developed for CHARMM22, did not perform well with the 36m version of the FF.

Finally, an amphiphilic, water‐soluble β‐undecapeptide has also been studied. According to the analysis of the intrachain H‐bond network, the initial H14 conformation is stable with our new FF extension, and interestingly, with the pristine CHARMM36m FF as well, mainly because the side‐chains – carefully parametrized in CHARMM36m – also contribute to the stability of the fold. The quick unfolding observed under the FF parametrization by Zhu et.al. is assumed to be caused by the incompatibility of the torsion parameters originally developed for CHARMM22.

These three examples demonstrate the importance of thorough parametrization and also the limitations of transferring parameters from a previous FF to another, more improved one.

Because the underlying CHARMM36m force field has been developed for a wide range of chemical compounds including proteins, nucleic acids, lipids, as well as small drug‐like molecules (with the aid of the compatible CGenFF), the extended version is expected to be able to model the interactions e. g. between β‐peptide foldamers and lipids, proving to be a useful tool in the study of the membrane‐activity of nonnatural peptidic foldamers, among others.

The force field files are available at https://bionano.ttk.mta.hu/software, as well as https://gitlab.com/awacha/charmm‐beta.ff. Documentation and user's manual is hosted at https://charmm‐betaff.readthedocs.io.

## Computational Methods

All *ab initio* calculations were done in accordance with the development of the CHARMM36m force field. Single point energies and analytic gradients (needed for the geometry optimization) of the four simple model peptides were calculated using all‐electron restricted MP2^57^ method with 6–31G(d) basis set^58^ with the Gaussian 09, Revision E.01 code.^59^ Zero charge and zero spin (multiplicity 1) were assumed for the molecules. A quadratically convergent (keyword “QC”) SCF procedure^60^ with the “Verytight” (threshold: 10^−8^) convergence criterion was used. Geometry optimizations were carried out using the Berny algorithm^61^ with GEDIIS^62^ in redundant internal coordinates.^63^

All MM and MD simulations presented herein were done using the 2018.2 version of the GROMACS suite.^64^, ^65^, ^66^, ^67^, ^68^, ^69^, ^70^, ^71^, ^72^ Details are given in the Supporting Information. For the least squares fitting procedure, a Python script was developed, using the Trust Region Reflective algorithm implemented in Scipy.^73^, ^74^, ^75^ The release dated July 2017. of the CHARMM36m FF has been used everywhere.

## Conflict of interest

The authors declare no conflict of interest.

## Supporting information

As a service to our authors and readers, this journal provides supporting information supplied by the authors. Such materials are peer reviewed and may be re‐organized for online delivery, but are not copy‐edited or typeset. Technical support issues arising from supporting information (other than missing files) should be addressed to the authors.

Supplementary
