# Supplementary material for: Improved Modeling of Peptidic Foldamers Using a Quantum Chemical Parametrization Based on Torsional Minimum Energy Path Matching
Source: Chempluschem. 2019 Jul 4;84(7):927–41. doi: 10.1002/cplu.201900180 (PMC6686720; doi:10.1002/cplu.201900180)
Supplement: Supplementary file 1 — Supplementary [file CPLU-84-927-s001.pdf]

CHEMPLUSCHEM

Supporting Information

© Copyright Wiley-VCH Verlag GmbH & Co. KGaA, 69451 Weinheim, 2019

Improved Modeling of Peptidic Foldamers Using a Quantum Chemical Parametrization Based on Torsional Minimum Energy Path Matching

András Wacha, Tamás Beke-Somfai,* and Tibor Nagy*©2019 The Authors. Published by Wiley-VCH Verlag GmbH & Co. KGaA.
This is an open access article under the terms of the Creative Commons Attribution License, which permits use, distribution and reproduction in any medium, provided the original work is properly cited.This article is part of the “Early Career Series”. To view the complete series, visit: .

1 Molecular dynamics simulation parameters

In the manuscript, whenever not noted explicitly, the following parameters were used:

Constraints: Lengths of the chemical bonds to hydrogen atoms were constrained using the LINCS algorithm. All other bond lengths were treated with the corresponding harmonic potentials.

Neighbour search algorithm: Verlet cut-off scheme with 1.2 nm cut-off distance, neighbour search is performed at every 20th step.

van der Waals forces: Cut-off algorithm with 1.2 nm cut-off distance and force switchoff after 1.0 nm, no long range dispersion corrections applied.

Coulomb potential:

In vacuum: Cut-off algorithm with 1.2 nm cut-off distance

With solvent: 4th order particle mesh Ewald (PME) method, using cubic interpolation with 0.16 nm^{-1} Fourier grid spacing

Temperature coupling: Velocity-rescaling (modified Berendsen) thermostat with 0.1 ps coupling time at 300 K reference temperature. The solvent (including ions) and the solute have been coupled to two separate thermostats in order to avoid the hot solvent/cold solute problem.

Pressure coupling: Parrinello-Rahman barostat at 1 bar, coupling with 5 ps time constant. The isothermal compressibility parameter of water has been taken ($4.6 \cdot 10^{-5}$ bar^{-1}).

Algorithm: Using the default leap-frog integrator ("md") of GROMACS, with 2 fs timesteps.

2 Preparation procedure of the H14 β-heptapeptide

In order to avoid exposing the molecule to thermal shocks, and to start the MD sampling runs from a well-solvated helical conformation, the following preparatory steps were carried out before commencing production simulations:

1. The chemical model of the peptide has been constructed in PyMOLSchrödinger, LLC 2015 in an extended conformation.
2. The peptide has been folded into the appropriate helix by adjusting the backbone torsion angles according to the work of Beke, Somlai, and Perczel 2006. We have further optimized the angles with a PyMOL script to set all hydrogen-acceptor distances nearly to 0.18 nm.
3. The topology for the molecule has been built by the "pdb2gmx" tool of GROMACS, and afterwards a dodecahedral box has been constructed around the geometrical center of the peptide with a minimum peptide-box edge distance of 2 nm.
4. A steepest descent energy minimization has been done on the structure. In this run, the hydrogen-acceptor distances were restrained to 0.18 nm with a 1000 kJ/mol harmonic potential.
5. Another energy minimization run has been made with the steepest descent algorithm, but this time the hydrogen bonds were unrestrained.
6. In order to assess the behaviour and the stability of the peptide, and to introduce velocities, an NVT simulation has been done *in vacuo* for 0.1 ns with 2 fs timesteps. Velocities were generated at the beginning in a random fashion from a Maxwell-Boltzmann distribution corresponding to 300 K. Throughout this run, the same restraint potential as above was applied to hydrogen bonds once again. After this, the simulation was continued for 10 ns with the same parameters, only the restraints on the H-bonds were lifted.
7. The end state of the H-bond restrained *in vacuo* NVT run has been used for further analysis. The peptide has been solvated in a pre-equilibrated methanol box (for the procedure see section §6). A single Cl^- ion has been added for neutrality. The whole solvent box (including the counterion) has been energy minimized with position restraint applied to all atoms of the peptide.
8. A velocity rescaling thermostat of 300 K has been turned on. The initial velocities were re-sampled from the corresponding Maxwell-Boltzmann distribution. Throughout the 100 ps time of this NVT ensemble simulation, the position restraints on the peptide has been upheld.
9. An NPT simulation using a Parrinello-Rahman barostat followed for 1 ns, also keeping the position restraints of the peptide.
10. At this point the position restraints were lifted, and the hydrogen bond length restraints were applied. The simulation was continued for 2 ns.
11. Finally, the hydrogen bond restraints were turned off linearly during a 0.2 ns NPT run.

3 Preparation procedure of the H14 β-heptapeptide started in an extended conformation

In order to assess the preference and stability of the 14-helical fold, we performed a simulation with a more "rough" preparation:

1. The chemical model of the peptide has been constructed in PyMOLSchrödinger, LLC 2015 in an extended conformation.
2. The topology for the molecule has been built by the "pdb2gmx" tool of GROMACS, and afterwards a dodecahedral box has been constructed around the geometrical center of the peptide with a minimum peptide-box edge distance of 2 nm.
3. A steepest descent energy minimization has been done on the structure. In contrast to the previous case (starting from the H14 fold), no restraints were placed on the H-bond distances, as there are no intrachain hydrogen bonds in this conformation, to start with.
4. The peptide has been solvated in a pre-equilibrated methanol box (for the procedure see section §6). A single Cl^- ion has been added for neutrality. The whole solvent box (including the counterion) has been energy minimized with position restraint applied to all atoms of the peptide.
5. A velocity rescaling thermostat of 300 K has been turned on. The initial velocities were re-sampled from the corresponding Maxwell-Boltzmann distribution. Throughout the 100 ps time of this NVT ensemble simulation, the position restraints on the peptide has been upheld.
6. An NPT simulation using a Berendsen barostat (1 ps coupling time) followed for 1 ns, also keeping the position restraints of the peptide.
7. Production runs were performed under the same conditions, but using the Parrinello-Rahman barostat (5 ps coupling time).

4 Preparation procedure of the hairpin β-hexapeptide

In order to avoid exposing the molecule to thermal shocks, and to start the MD sampling runs from a well-solvated, stable hairpin conformation, the following preparatory steps were carried out before commencing production simulations:

1. The chemical model of the peptide has been constructed in PyMOLSchrödinger, LLC 2015 in an extended conformation.
2. The peptide has been folded into an approximate hairpin conformation by adjusting the backbone torsion angles by hand.
3. The topology for the molecule has been built by the "pdb2gmx" tool of GROMACS, and afterwards a dodecahedral box has been constructed around the geometrical center of the peptide with a minimum peptide-box edge distance of 2 nm.
4. A steepest descent energy minimization has been done on the structure with position restraints (harmonic potential, 100000 kJ/mol/nm) applied to the backbone atoms, in order remove residual strains induced by non-optimal side-chain conformations.
5. The energy minimization was continued with the restraint potential on the backbone atoms lifted, while the hydrogen-acceptor distances of the three intra-chain hydrogen bonds responsible for the hairpin conformation were restrained to 0.18 nm with a 1000 kJ/mol harmonic potential in order to avoid premature unfolding of the structure.
6. Finally, after residual strains in the side-chains and backbone were thus removed, an unrestrained energy minimization run has been made.
7. The end state of the last energy minimization step has been used for further processing. The peptide has been solvated in a pre-equilibrated methanol box (for the procedure see section §6). Energy minimization has been done on the solvent box with 1000 kJ/mol/nm harmonic position restraints applied to all atoms of the peptide.
8. A velocity rescaling thermostat of 300 K has been turned on. The initial velocities were sampled from the Maxwell-Boltzmann distribution corresponding to 300 K. Throughout the 100 ps time of this NVT ensemble simulation, the position restraints on the peptide has been upheld.
9. An NPT simulation using a Parrinello-Rahman barostat followed for 1 ns, also keeping the position restraints of the peptide.
10. At this point the position restraints were lifted, and the hydrogen bond length restraints were applied, this time restrained to the actual distances at the start of the simulation. The simulation was continued for 1 ns.
11. Finally, the hydrogen bond restraints were turned off linearly during a 2 ns NPT run.

5 Preparation procedure of the amphiphilic H14 β-undecapeptide

Observing the folding of the "VALXVAL" heptapeptide when started from an extended conformation, we became more confident and started this simulation – although from the helical state – using a less gentle approach than in the case of the heptapeptide:

1. The chemical model of the peptide has been constructed in PyMOLSchrödinger, LLC 2015 in an extended conformation, and folded into the H14M conformation by adjusting the backbone torsion angles to the values published by Beke, Somlai, and Perczel 2006. No other structural refinement/manipulation has been done.

2. The topology for the molecule has been built by the "pdb2gmx" tool of GROMACS, and afterwards a dodecahedral box has been constructed around the geometrical center of the peptide with a minimum peptide-box edge distance of 1.5 nm.

3. A steepest descent energy minimization has been done on the structure, with restraining forces applied to the backbone atoms, to make the side-chains relax.

4. Energy minimization was continued with lifting the backbone position-restraints.

5. The peptide has been solvated in a water box (using the spc216.gro box supplied with GROMACS). NaCl ions were added in a concentration corresponding to 0.15 mol/dm^3. The whole solvent box (including the ions) has been energy minimized with position restraint applied to all atoms of the peptide.

6. A velocity rescaling thermostat of 300 K has been turned on. The initial velocities were re-sampled from the corresponding Maxwell-Boltzmann distribution. Throughout the 3 ns time of this NVT ensemble simulation, the position restraints on the peptide has been upheld.

7. An NPT simulation using a Berendsen barostat (1 ps coupling time) followed for 10 ns, also keeping the position restraints of the peptide.

8. Production runs were performed under the same conditions, but using the Parrinello-Rahman barostat (5 ps coupling time).

6 Construction of a methanol solvent box

For the solvation of the "VALXVAL" heptapeptide and the "HP6" hexapeptide, a methanol box has been constructed according to the following procedure. The methanol model of CHARMM36m has been used. First, the geometry of a single methanol molecule has been created. After placing it into a $2 \times 2 \times 2$ nm^3 box, a short steepest descent energy minimization followed, then the resulting structure has been multiplied by the `genconf` subprogram of GROMACS, yielding a cubic box of 10 nm edge length, containing 125 randomly oriented methanol molecules. Next, a NPT simulation at 300 K and 100 bar pressure has been applied to liquify the methanol. Finally, the resulting structure has been equilibrated at 300 K (Velocity rescaling thermostat, 0.1 ps coupling time) and 1 bar (isotropic Berendsen barostat, 1 ps coupling time, $12 \cdot 10^5$ bar^{-1} isotherm compressibility) for 10 ns.

7 Technical details on the derivation of the new atom types

As stated in the main text, the new atom types for the α and β carbons of the β-peptide backbone are CTA1, CTA2, CTB1 and CTB2. These are basically clones of CT1 and CT2 (aliphatic sp^3 carbons in proteins with 1 or 2 hydrogens, respectively) in the original CHARMM36 force field. All nonbonded and some bonded (bond, angle, Urey-Bradley and improper dihedral) parameters involving the original atom types have been replicated with the new atom types.

Proper dihedral types were handled differently. First dihedral types for all optimizable dihedrals of the four diamides used for parametrization were created with placeholders for the actual values determined later by the fitting procedure outlined in the main text. The molecular topologies of acyclic β-amino acid residues with all possible combination of the 19 proteinogenic side-chains (excluding proline) were constructed, and the missing angle and bond parameters were determined from chemical analogies.

The missing dihedral types were determined by the following three procedures in order:

1. Dihedral types corresponding to the backbone were replicated to allow for different side-chains or terminal groups. As an example, four additional copies of $\varphi_2^{(3)}$ (corresponding to atom types C, NH1, CTA1, CT3) have been made by replacing CT3 with CT1, CT2, CT2A and CS

2. An attempt was made to derive the still missing dihedral types using the ancestor atom types, i.e. CTA2, C, NH1, CT3 from CT2, C, NH1, CT3. If no exact match was found, wildcard parameters (e.g. X, CT1, CT2, X) have been also considered.

3. Finally, chemical analogies were used to determine the rest of missing dihedral parameters and those angle and Urey-Bradley types that were not generated by the cloning procedure.

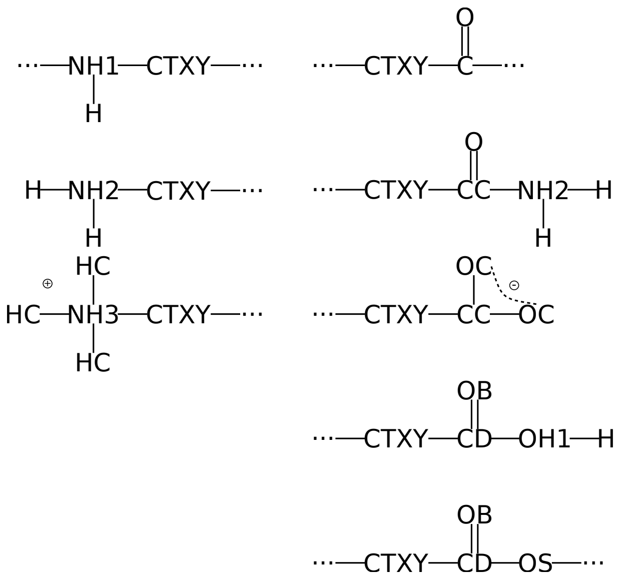

Figure S1: CHARMM36 atom types for various termini. "CTXY" denotes the atom types introduced by this work for the α and backbone β-carbons of the β-amino acids (i.e. CTA1, CTA2, CTB1 and CTB2).

8 Generating β-residue topologies

As an aid to the prospective users and for ensuring the completeness of the parameter set, we have constructed the topologies of acyclic β-amino acids with all possible combinations of the 19 proteinogenic side-chains (with additional variations in the protonation state, in total 25 variations). Altogether 625 residues (one side-chainless, 25 β^2, 25 β^3 and 25×25 25 $\beta^{2,3}$) were generated. Partial charges and charge groups were assigned by following the rules of the original amino acid residues in the CHARMM force field:

- The atoms C, O, N and H in the amide bond have a partial charge of 0.51, -0.51, -0.47 and 0.31, respectively.
- All other hydrogens have a 0.09 partial charge.
- N, H, C_β and all the hydrogens attached to C_β belong to the same charge group. Therefore if there is a side-chain on the β carbon, the partial charge on C_β is 0.07, otherwise -0.02.
- C_α and its hydrogens form a charge group by themselves, thus the partial charge on C_α is -0.09 if there is a side-chain on it, else -0.180 to compensate for the two hydrogens.
- Partial charges of side-chain atoms were set the same as of the corresponding atoms in the original residue topologies. Charge grouping has also been taken without modification.

The atom naming in the residues follows the rules in the CHARMM force field. For β^3-amino acids the naming is shifted in the Greek alphabet, e.g. the first carbon of the side chain (CB) becomes CG. For β^2 and $\beta^{2,3}$-amino acids the β-carbon in the backbone becomes CB1, and the first atom on the β^2-side-chain CB2. In the enumeration of atoms with the same distance from the amide carbon (having the same Greek letter) atoms in the β^3-side-chain take precedence, otherwise the naming conventions of the original amino-acid side-chains are followed.

9 How to use the extended force field

Obtaining force field files

The force field files are available, presently only in GROMACS format under at https://gitlab.com/awacha/charmm-beta.ff. A more detailed documentation is hosted at https://charmm-betaff.readthedocs.io/en/latest/.

Residue naming

The FF extension comes with a pre-generated list of β-amino acid residue topologies, readily usable for assigning FF parameters for atom types and interactions to the user-supplied geometry. Residue naming is according to the rule:

`B2<s>` for β^2-, `B3<s>` for β^3- and `B0<s><s>` for $\beta^{2,3}$-substituted amino-acids, where `<s>` is the designation of the side-chain based on the single letter code of proteinogenic amino-acid side-chains, which can be either one or two characters long. The possible values are enumerated in Table S1. Note that because CHARMM limits residue names in 4 characters, residues which would have longer names according to the above rules have not been generated. And as the derived backbone torsion parameters are invariant for stereoisomerism, no distinction is made in the residue naming between *S* and *R* absolute conformations.

A few examples:

Abbreviation	Description
A	Alanine
C	Cysteine
CM	Anionic cysteine
D	Aspartate (ionic)
DH	Aspartic acid (protonated)
E	Glutamic acid
EH	Protonated glutamic acid
F	Phenyl-alanine
G	Glycine (i.e. no side-chain)
HD	Histidine protonated on N_δ
HE	Histidine protonated on N_ε
HH	Histidine protonated on both N_δ and N_ε
I	Isoleucine
K	Lysine
KN	Neutral lysine
L	Leucine
M	Methionine
N	Asparagine
Q	Glutamine
R	Arginine
S	Serine
T	Threonine
V	Valine
W	Tryptophan
Y	Tyrosine

Table S1: Abbreviations of the proteinogenic side-chains used in the naming of β-peptide residues.

(*S*)-β^2-hK: B2K

(*R*)-β^3-hV: B3V

(2*S*,3*R*)-β^{2,3}-h(2A,3V): B0AV (the '0' character is included in order to avoid name clashes with already existing residues)

βA: BALA (i.e. side-chainless, achiral β-amino acid)

Coordinates

According to this naming scheme, the longest residue name can be seven characters long. Therefore such a file format must be chosen for the storage of geometrical information, which can accommodate this long residue names, e.g. the GROMOS96 (*.g96) format in case of GROMACS and the expanded CRD format in CHARMM c31a1 and later. For creating the geometries themselves (and labeling them in accordance with the above described rules), one possibility is to use the `pmlbeta` PyMOL extension developed by one of the authors of the present work (obtainable at https://gitlab.com/awacha/pmlbeta).

GROMACS-specific information

Having the correctly labelled β-peptide geometry at hand, one can use the pdb2gmx utility of GROMACS (tested with versions 2016 and upwards) to create the topology. The force field directory (charmm36-jul2017-beta.ff) should either be put in the `$GMXDATA/top` directory or the current working directory for the simulations. Likewise, the `residuetypes.dat` file must be copied to `$GMXDATA/top` or put in the current working directory in order to make the GROMACS tools recognize β-peptide residues as proteins.

If not instructed otherwise, the optimized parameters for the β-peptide backbone torsion angles will be used. The parameters of the pristine CHARMM36m force field (FFc36 in the main text) or that of Zhu et al. (2010) can be used if BETA_ORIGINAL or BETA_ZHUETAL is defined in the MDP file (with `define = -DBETA_ORIGINAL` or `define = -DBETA_ZHUETAL` options).

10 Choosing the RMSD threshold for the calculation of Gibbs free energy of folding

In the work of Huang, Lin, and Gunsteren (2011), the following formula was used for determining the free Gibbs free energy of folding:

$$\Delta G_{\mathrm{folding}} = -k_B T \ln \left(p_{\mathrm{folded}} / p_{\mathrm{unfolded}}\right),$$

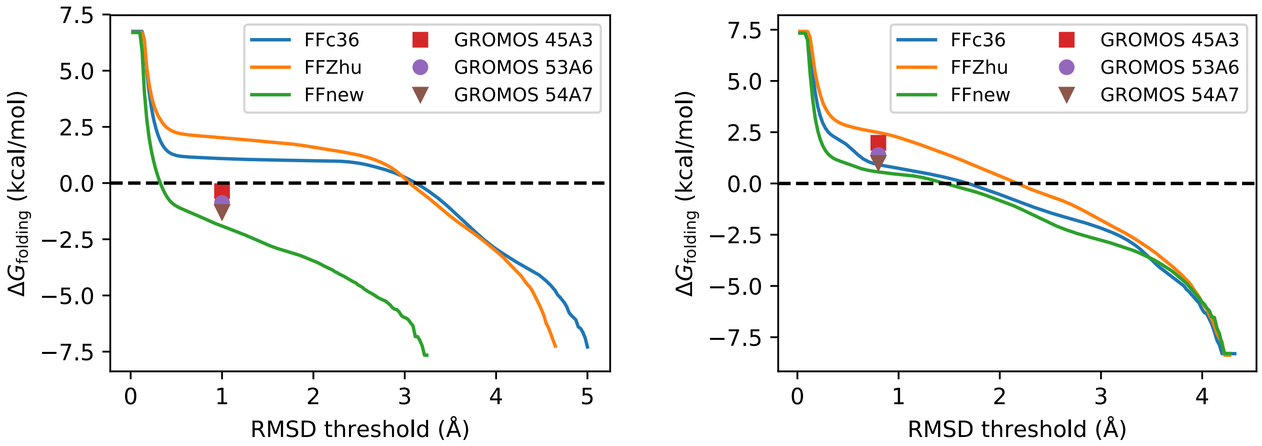

Figure S2: Dependence of the Gibbs free energy of folding on the RMSD threshold between the folded and unfolded states for the VALXVAL heptapeptide (left) and the HP6 hexapeptide (right). The dashed line corresponds to $\Delta G_{\text{folding}} = 0$. The single marker points correspond to the values reported by Huang, Lin, and Gunsteren (2011) for the same peptides with various versions of the GROMOS force field.

where p_{folded} or p_{unfolded} are the probabilities that the system is in the folded or the unfolded state, respectively. In order to classify the frames of the trajectory into these two states, they have selected a reference structure (conveniently the initial helical structure in which the peptide was prepared before the simulation) and categorized all frames nearer than a given threshold to this state in terms of root-mean-square deviation of backbone atom positions as "folded", and all the others "unfolded". For the value of this threshold they have chosen 1 Å in case of the VALXVAL peptide and 0.8 Å for HP6, based upon the analysis of the RMSD distributions of the trajectories. In Figure S2 the dependence of the calculated value of $\Delta G_{\text{folding}}$ is shown as a function of the RMSD threshold. The values published by Huang et al. are also shown, albeit they have been obtained at a different temperature (at 340 K, in contrast to our 300 K). According to the graphs, FFnew, our proposed extension to the CHARMM36m force field always predicts a more stable folded structure than the pristine CHARMM36 force field (FFc36) or its extension by Zhu et al. (2010).

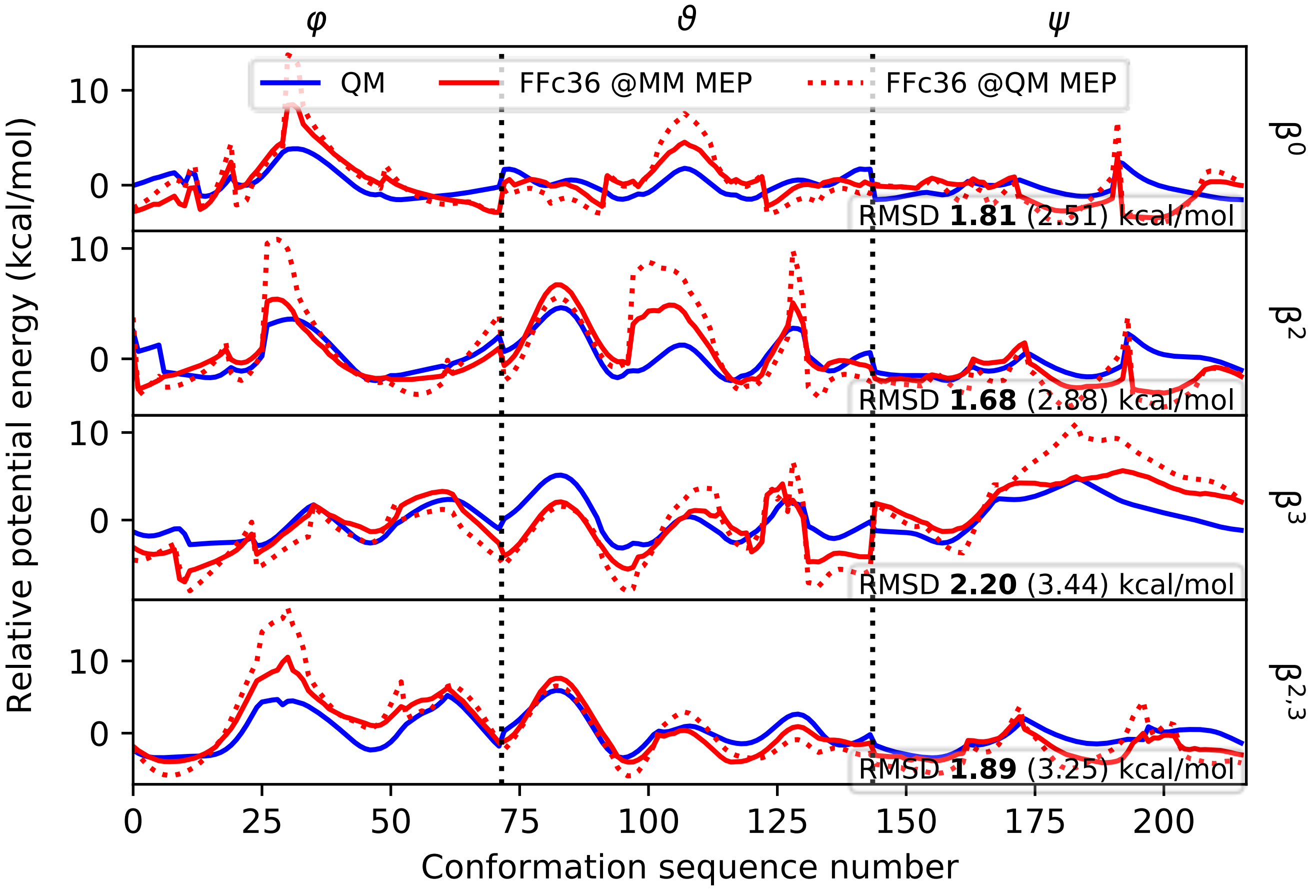

Figure S3: Fit of the QM (solid blue line) and MM (red lines) potential energies of the Ac-βAla-NHMe, Ac-(S)β^{2}hAla-NHMe, Ac-(S)β^{3}hAla-NHMe and Ac-(2S,3S)β^{2,3}-h(2Ala,3Ala)-NHMe peptides. The MM potential energies were calculated using the unmodified CHARMM36m force field on the MM MEP (solid red line, RMSD in boldface) and the QM MEP (dotted red line, RMSD in regular text).

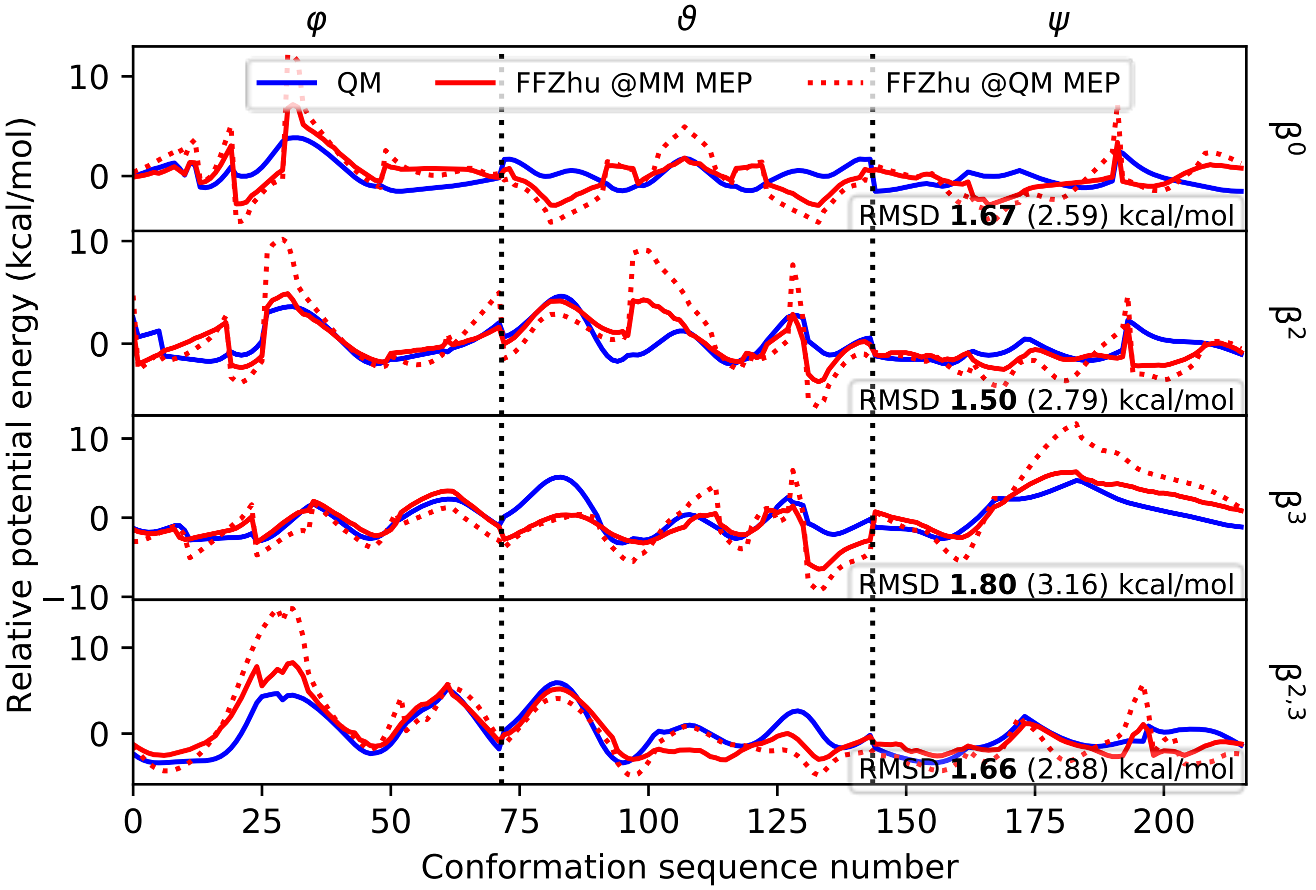

Figure S4: Fit of the QM (solid blue line) and MM (red lines) potential energies of the Ac-βAla-NHMe, Ac-(S)β^2hAla-NHMe, Ac-(S)β^3hAla-NHMe and Ac-($2S$,$3S$)$\beta^{2,3}$-h(2Ala,3Ala)-NHMe peptides. The MM potential energies were calculated using the CHARMM36m force field extended with the parameters proposed by Zhu et al. (2010), on the MM MEP (solid red line, RMSD in boldface) and the QM MEP (dotted red line, RMSD in regular text).

11 Supporting figures

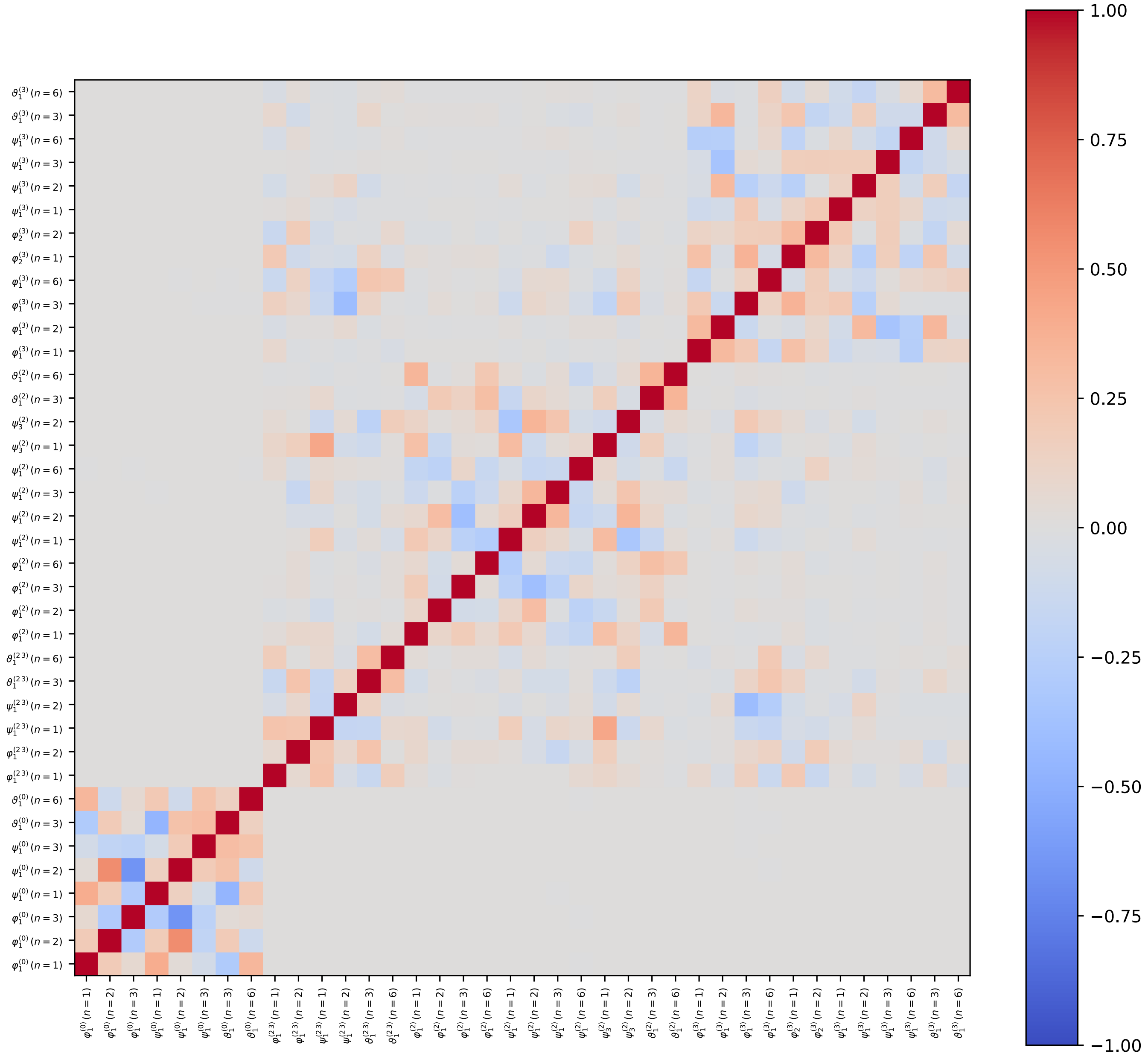

Figure S5: Correlation matrix of the fitted values of the final set of independent half barrier height parameters.

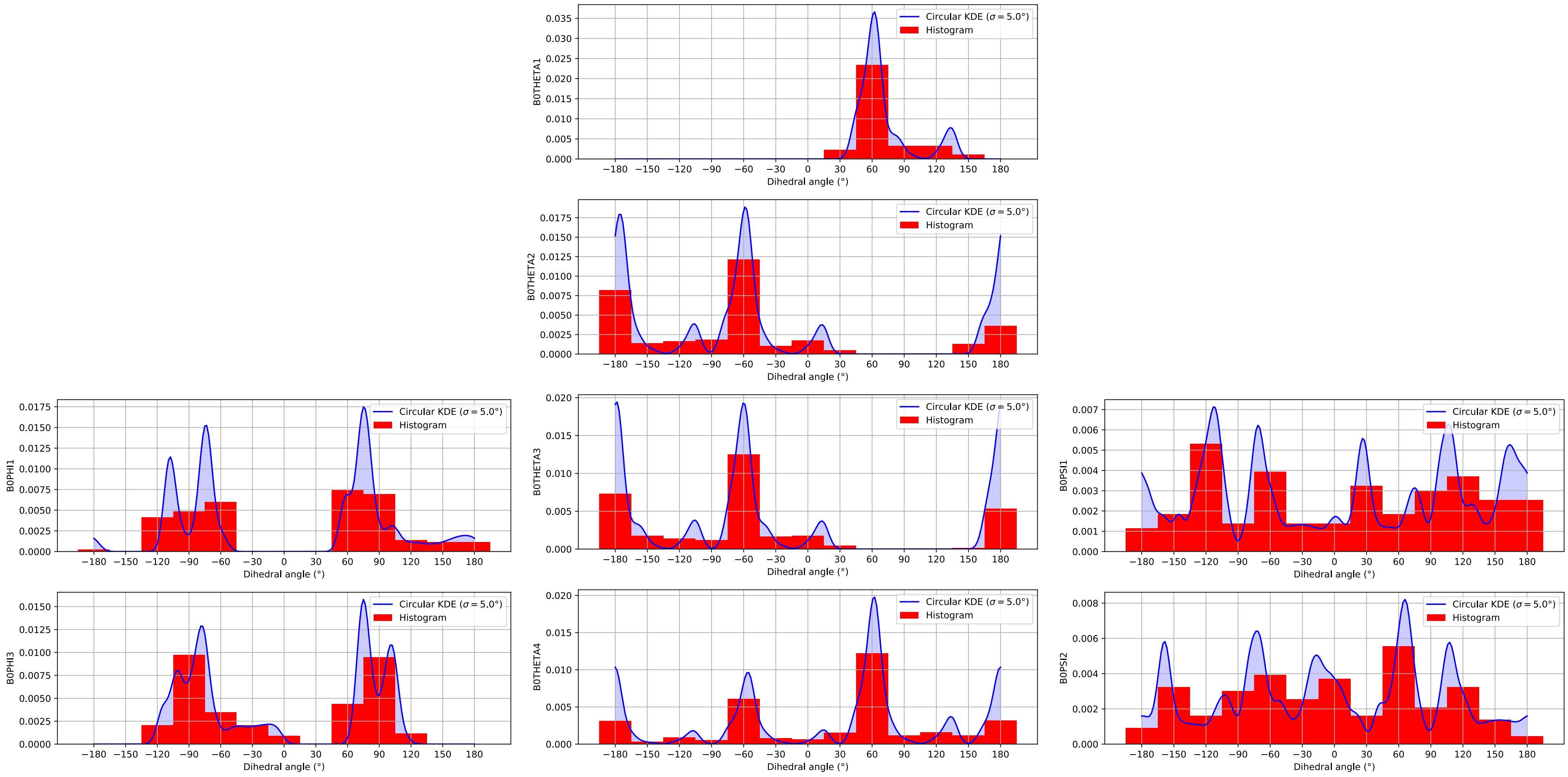

Figure S6: Torsion angle histograms calculated from the QM torsion scans of the Ac-βA-NHMe diamide. Circular kernel density estimations were done with a von Mises kernel of 5° half width.

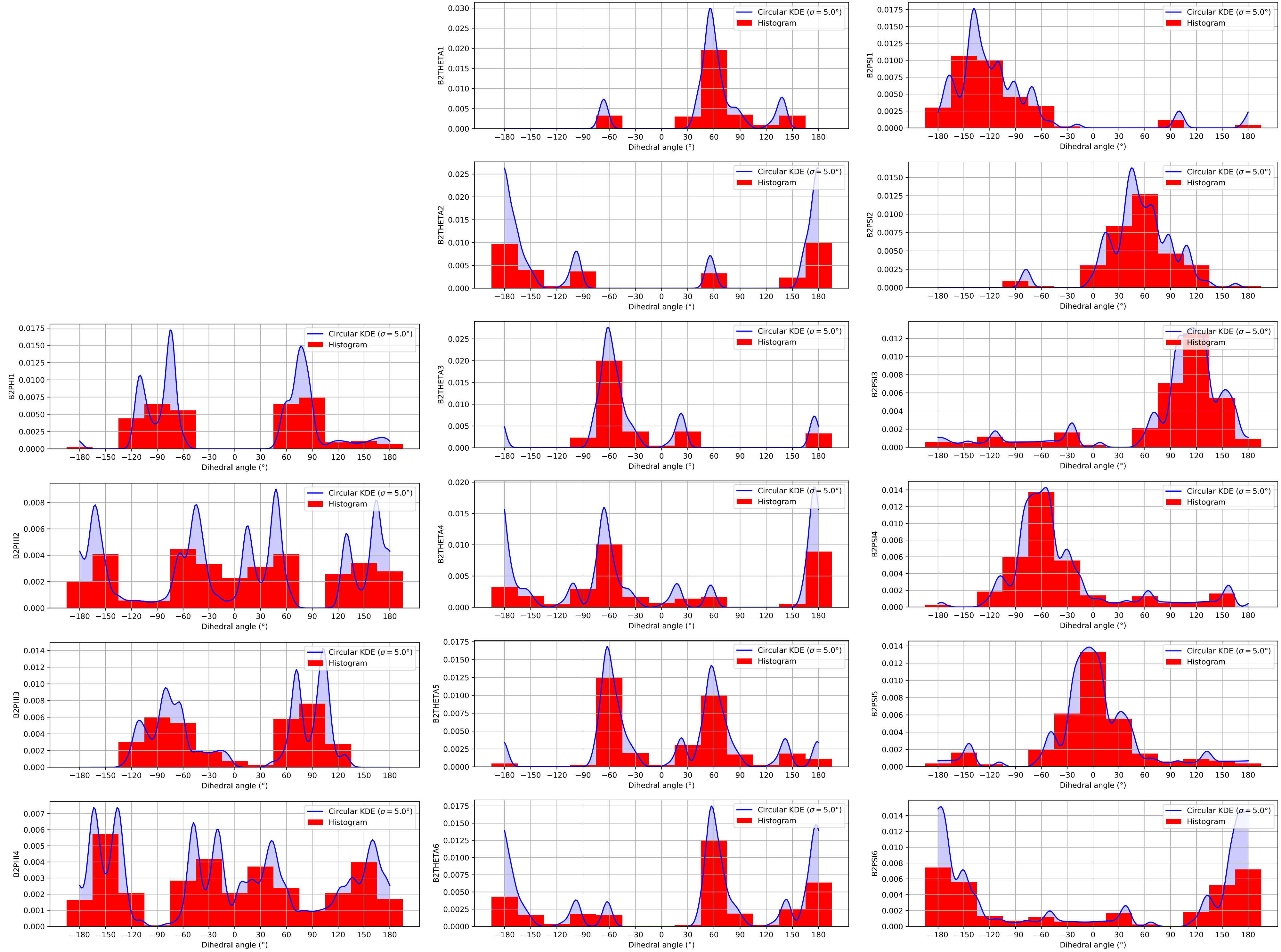

Figure S7: Torsion angle histograms calculated from the QM torsion scans of the Ac-(S)β^{2}hA-NHMe diamide. Circular kernel density estimations were done with a von Mises kernel of 5° half width.

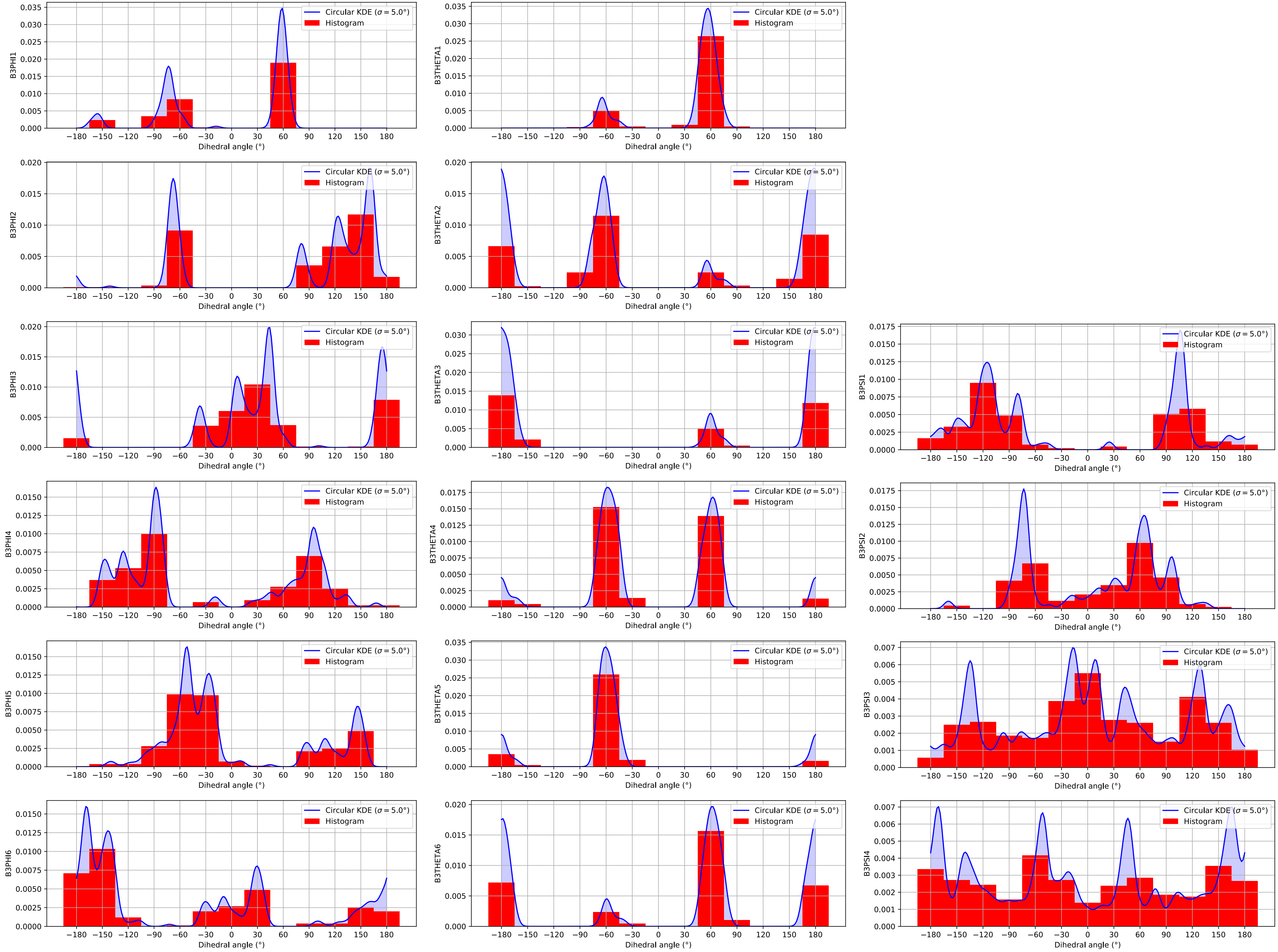

Figure S8: Torsion angle histograms calculated from the QM torsion scans of the Ac-$(S)\beta^3$hA-NHMe diamide. Circular kernel density estimations were done with a von Mises kernel of 5° half width.

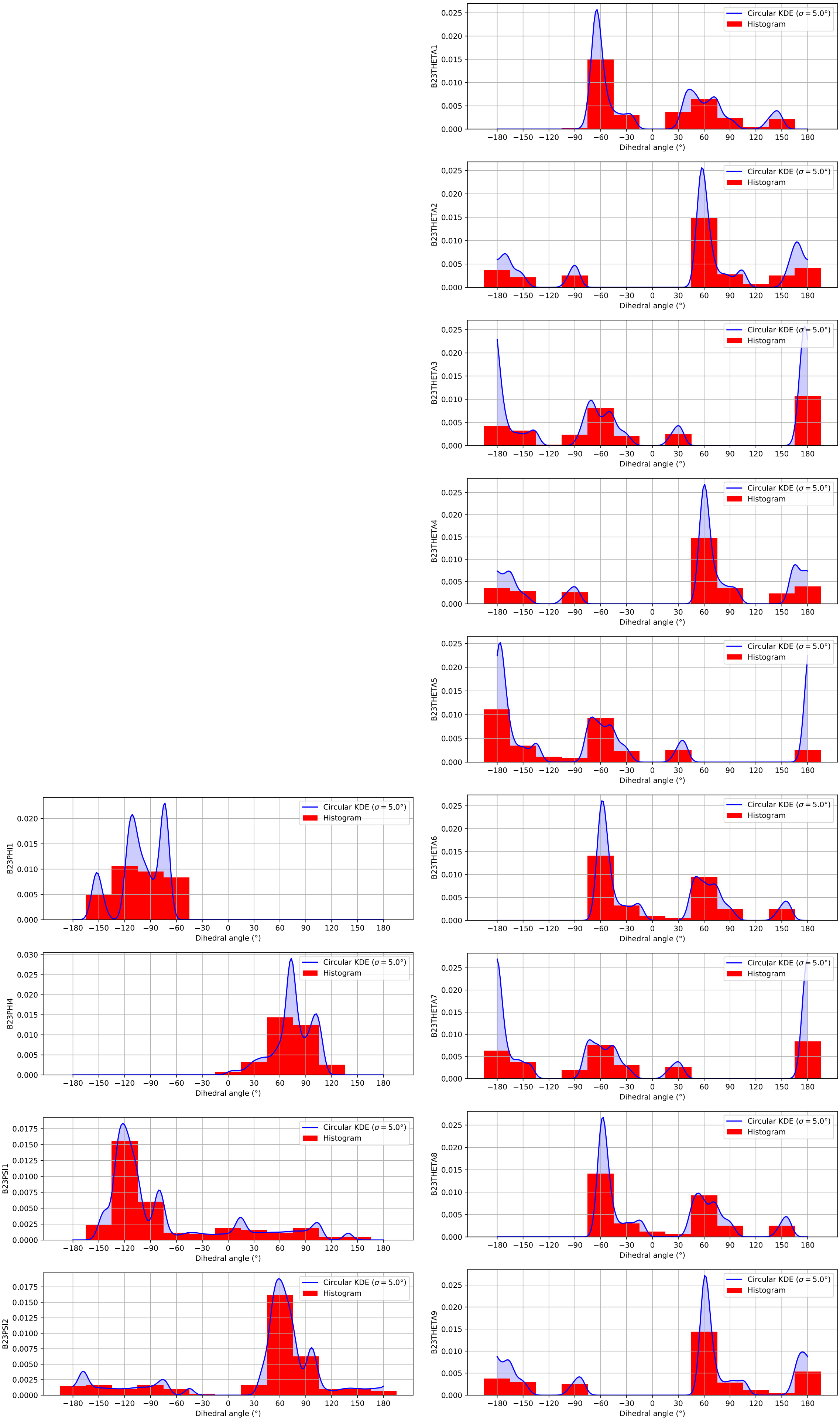

Figure S9: Torsion angle histograms calculated from the QM torsion scans of the Ac-(2S,3S)$\beta^{2,3}$h(2A,3A)-NHMe diamide. Circular kernel density estimations were done with a von Mises kernel of 5° half width.

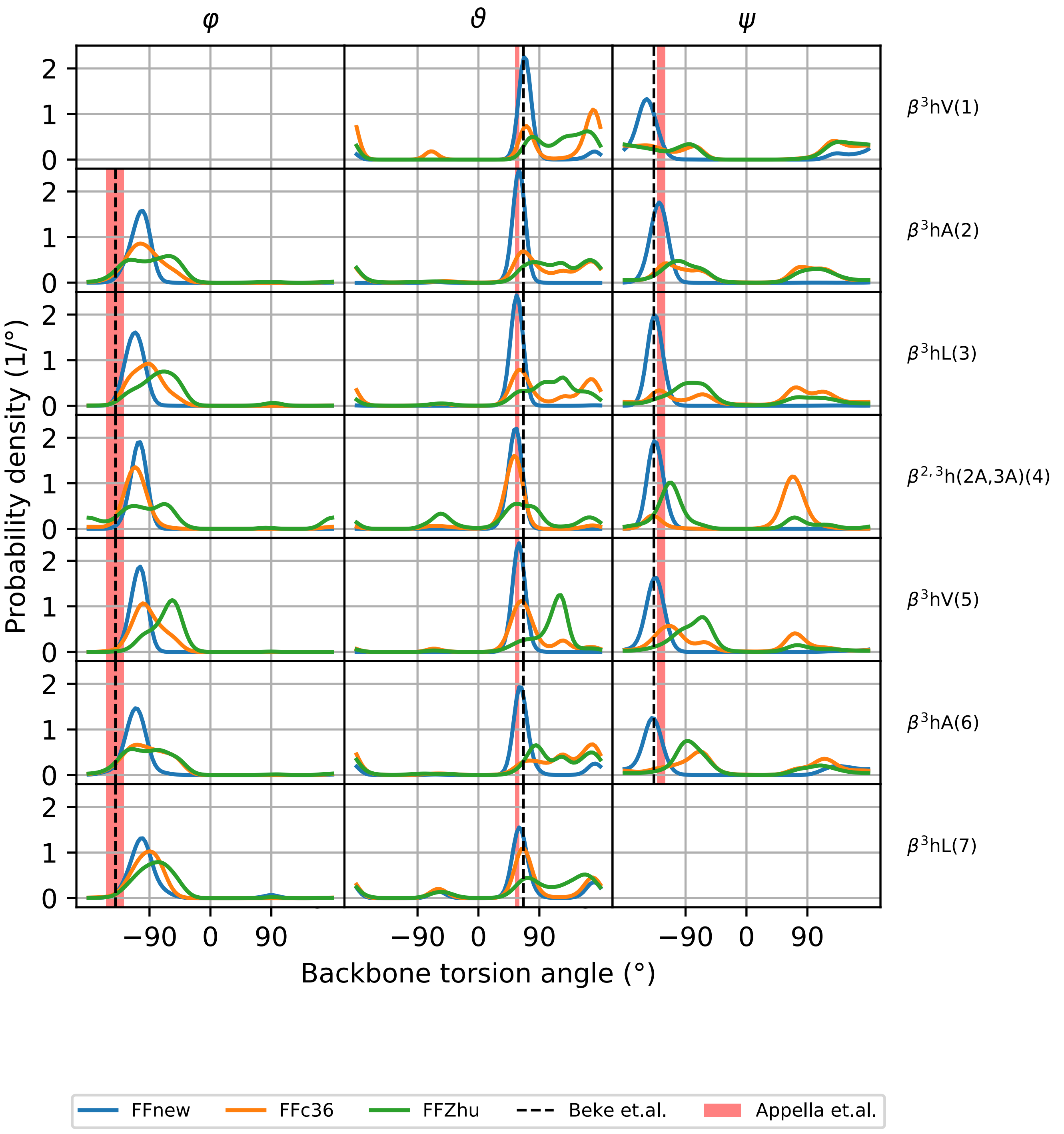

Figure S10: Kernel density estimation (von Mises kernel, 5° half width) of backbone torsion angles in the MD simulation of the "VALXVAL" heptapeptide.

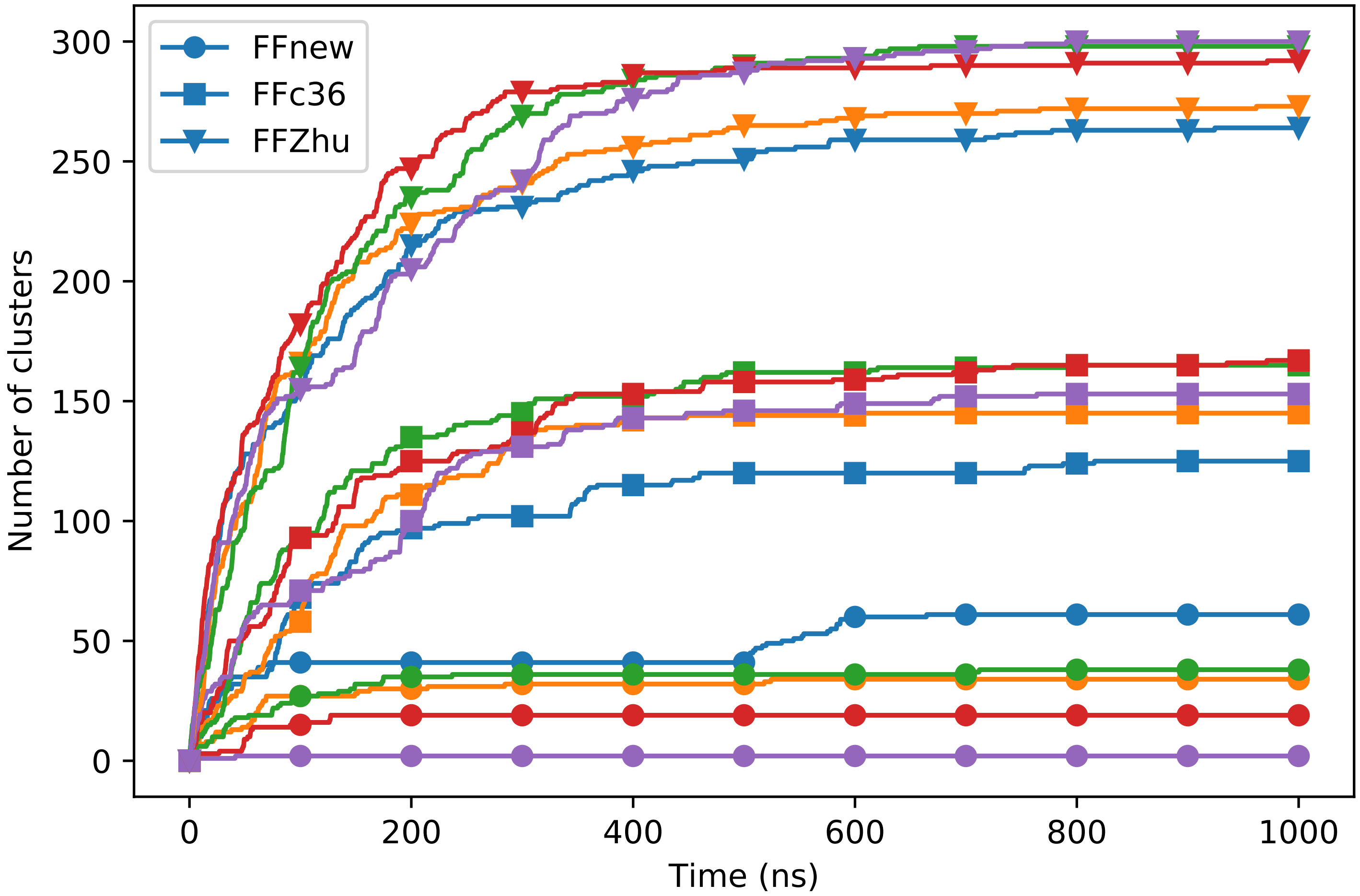

Figure S11: Number of clusters required for covering the trajectories of individual runs of the VALXVAL heptapeptide, started from an elongated conformation. Only clusters needed to cover 95% of the whole trajectory are taken into account. For clarity, only every 1000th point has been marked.

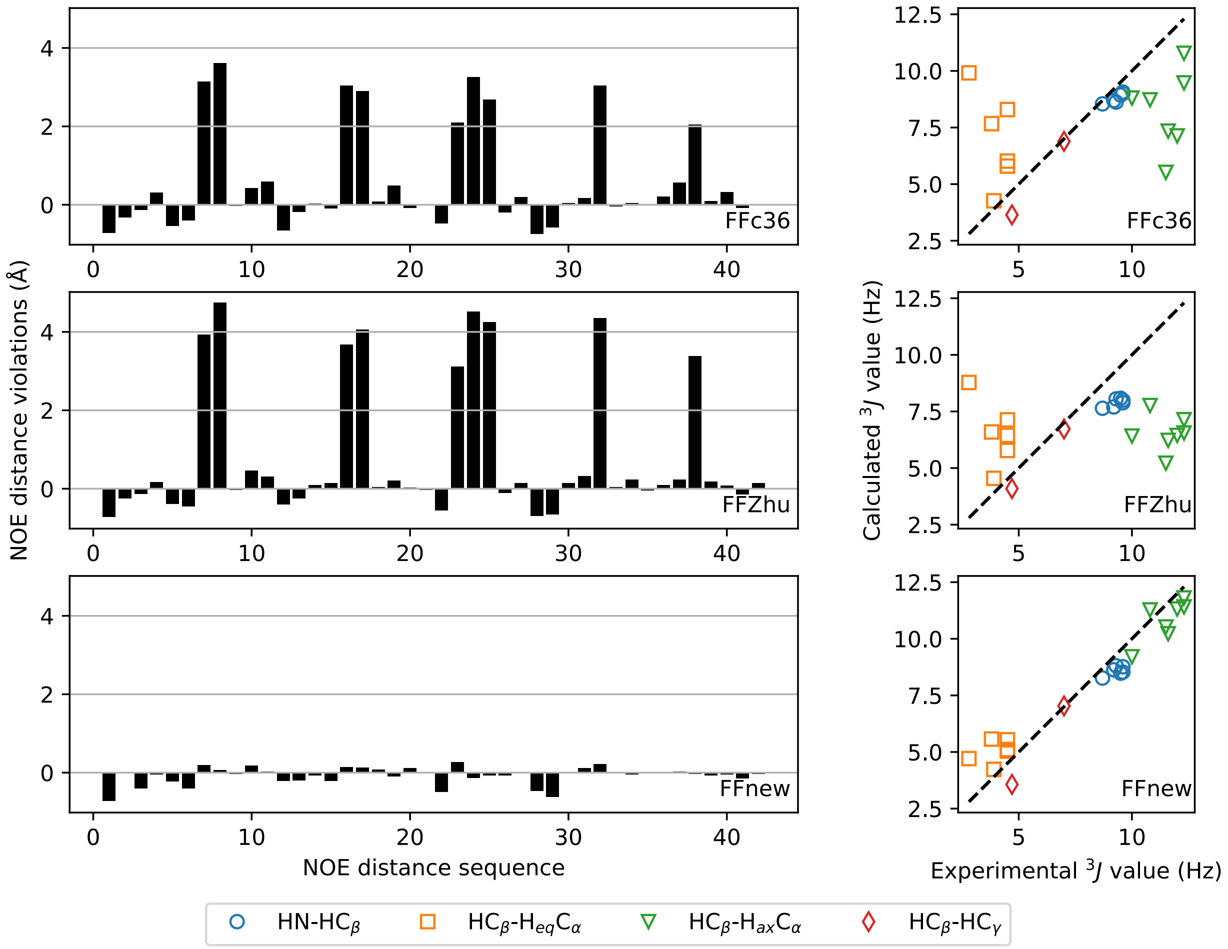

Figure S12: NOE distance violations (left) and ^3J-coupling constants (right) of the heptapeptide according to the original CHARMM36m force field (FFc36, top), the parametrization proposed by Zhu et al. (FFZhu, center) and this work (FFnew, bottom). All available frames of the trajectories were used for the averaging. The simulation runs were started from the extended/elongated conformation

12 Supporting tables

Table S2: Potential well positions for different multiplicities and offset phases

n	$\chi_0 = 0°$	$\chi_0 = 180°$
1	180°	0°
2	90°, 270°	0°, 180°
3	60°, 180°, 300°	0°, 120°, 240°
6	30°, 90°, 150°, 210°, 270°, 330°	0°, 60°, 120°, 180°, 240°, 300°

Table S3: Torsion angles and atom types

β⁰-peptide		β²-peptide		β³-peptide		$\beta^{2,3}$-peptide	
angle	atom types	angle	atom types	angle	atom types	angle	atom types
$\varphi_1^{(0)}$	C, NH1, CTB2, CTA2	$\varphi_1^{(2)}$	C, NH1, CTB2, CTB1	$\varphi_1^{(3)}$	C, NH1, CTA1, CTA2	$\varphi_1^{(2,3)}$	C, NH1, CTA1, CTB1
$\varphi_2^{(0)}$	C, NH1, CTB2, HB2	$\varphi_2^{(2)} = \varphi_2^{(0)}$	C, NH1, CTB2, HB2	$\varphi_2^{(3)}$	C, NH1, CTA1, CT3	$\varphi_2^{(2,3)} = \varphi_2^{(3)}$	C, NH1, CTA1, CT3
$\varphi_3^{(0)}$	H, NH1, CTB2, CTA2	$\varphi_3^{(2)}$	H, NH1, CTB2, CTB1	$\varphi_3^{(3)}$	C, NH1, CTA1, HB1	$\varphi_3^{(2,3)} = \varphi_3^{(3)}$	C, NH1, CTA1, HB1
$\varphi_4^{(0)}$	H, NH1, CTB2, HB2	$\varphi_4^{(2)} = \varphi_4^{(0)}$	H, NH1, CTB2, HB2	$\varphi_4^{(3)}$	H, NH1, CTA1, CTA2	$\varphi_4^{(2,3)}$	H, NH1, CTA1, CTB1
				$\varphi_5^{(3)}$	H, NH1, CTA1, CT3	$\varphi_5^{(2,3)} = \varphi_5^{(3)}$	H, NH1, CTA1, CT3
				$\varphi_6^{(3)}$	H, NH1, CTA1, HB1	$\varphi_6^{(2,3)} = \varphi_6^{(3)}$	H, NH1, CTA1, HB1
$\vartheta_1^{(0)}$	NH1, CTB2, CTA2, C	$\vartheta_1^{(2)}$	NH1, CTB2, CTB1, C	$\vartheta_1^{(3)}$	NH1, CTA1, CTA2, C	$\vartheta_1^{(2,3)}$	NH1, CTA1, CTB1, C
$\vartheta_2^{(0)}$	NH1, CTB2, CTA2, HB2	$\vartheta_2^{(2)}$	NH1, CTB2, CTB1, CT3	$\vartheta_2^{(3)}$	NH1, CTA1, CTA2, HB2	$\vartheta_2^{(2,3)}$	NH1, CTA1, CTB1, CT3
$\vartheta_3^{(0)}$	HB2, CTB2, CTA2, C	$\vartheta_3^{(2)}$	NH1, CTB2, CTB1, HB1	$\vartheta_3^{(3)}$	CT3, CTA1, CTA2, C	$\vartheta_3^{(2,3)}$	NH1, CTA1, CTB1, HB1
$\vartheta_4^{(0)}$	HB2, CTB2, CTA2, HB2	$\vartheta_4^{(2)}$	HB2, CTB2, CTB1, C	$\vartheta_4^{(3)}$	CT3, CTA1, CTA2, HB2	$\vartheta_4^{(2,3)}$	CT3, CTA1, CTB1, C
		$\vartheta_5^{(2)}$	HB2, CTB2, CTB1, CT3	$\vartheta_5^{(3)}$	HB1, CTA1, CTA2, C	$\vartheta_5^{(2,3)}$	CT3, CTA1, CTB1, CT3
		$\vartheta_6^{(2)}$	HB2, CTB2, CTB1, HB1	$\vartheta_6^{(3)}$	HB1, CTA1, CTA2, HB2	$\vartheta_6^{(2,3)}$	CT3, CTA1, CTB1, HB1
						$\vartheta_7^{(2,3)}$	HB1, CTA1, CTB1, C
						$\vartheta_8^{(2,3)}$	HB1, CTA1, CTB1, CT3
						$\vartheta_9^{(2,3)}$	HB1, CTA1, CTB1, HB1
$\psi_1^{(0)}$	CTB2, CTA2, C, NH1	$\psi_1^{(2)}$	CTB2, CTB1, C, NH1	$\psi_1^{(3)}$	CTA1, CTA2, C, NH1	$\psi_1^{(2,3)}$	CTA1, CTB1, C, NH1
$\psi_2^{(0)}$	CTB2, CTA2, C, O	$\psi_2^{(2)}$	CTB2, CTB1, C, O	$\psi_2^{(3)}$	CTA1, CTA2, C, O	$\psi_2^{(2,3)}$	CTA1, CTB1, C, O
$\psi_3^{(0)}$	HB2, CTA2, C, NH1	$\psi_3^{(2)}$	CT3, CTB1, C, NH1	$\psi_3^{(3)} = \psi_3^{(0)}$	HB2, CTA2, C, NH1	$\psi_3^{(2,3)} = \psi_3^{(2)}$	CT3, CTB1, C, NH1
$\psi_4^{(0)}$	HB2, CTA2, C, O	$\psi_4^{(2)}$	CT3, CTB1, C, O	$\psi_4^{(3)} = \psi_3^{(0)}$	HB2, CTA2, C, O	$\psi_4^{(2,3)} = \psi_4^{(2)}$	CT3, CTB1, C, O
		$\psi_5^{(2)}$	HB1, CTB1, C, NH1			$\psi_5^{(2,3)} = \psi_5^{(2)}$	HB1, CTB1, C, NH1
		$\psi_6^{(2)}$	HB1, CTB1, C, N			$\psi_6^{(2,3)} = \psi_6^{(2)}$	HB1, CTB1, C, O

	H atoms		NOE distance violations (Å)		
			FFc36	FFZhu	FFnew
1	H-N(1)	H-C_β(1)	-0.72	**-0.73**	**-0.71**
2	H-C_β(1)	H_{ax}-C_α(1)	-0.25	-0.14	0.09
3	H-C_β(1)	H_{eq}-C_α(1)	-0.21	-0.14	-0.44
4	H-N(2)	H_{ax}-C_α(1)	0.10	0.10	-0.10
5	H-N(2)	H_{eq}-C_α(1)	-0.33	-0.33	-0.13
6	H-N(2)	H-C_β(2)	-0.41	-0.45	-0.39
7	H-N(2)	H-C_β(4)	2.28	3.49	-0.02
8	H-N(2)	H-C_β(5)	2.15	3.89	-0.16
9	H-C_β(2)	H-C_γ(2)	-0.03	-0.04	-0.03
10	H-C_β(2)	H_{eq}-C_α(2)	0.39	0.47	0.13
11	H-N(3)	H_{ax}-C_α(2)	0.36	0.31	-0.05
12	H-N(3)	H_{eq}-C_α(2)	-0.47	-0.41	0.03
13	H-N(3)	H-C_β(3)	-0.20	-0.27	-0.20
14	H-N(3)	H_{ax}-C_α(3)	0.06	0.08	-0.14
15	H-N(3)	H-N(4)	0.19	0.10	-0.31
16	H-N(3)	H-C_β(5)	2.06	3.30	-0.11
17	H-N(3)	H-C_β(6)	**2.44**	3.46	-0.04
18	H-C_β(3)	H-C_δ(3)	0.09	0.10	0.14
19	H-N(4)	H_{ax}-C_α(3)	0.37	0.20	-0.15
20	H-N(4)	H_{eq}-C_α(3)	-0.22	0.04	**0.26**
21	H-N(4)	H-C_β(4)	0.02	-0.03	0.02
22	H-N(4)	H-C_γ(4)	-0.49	-0.56	-0.47
23	H-N(4)	H-C_β(6)	2.20	2.95	0.09
24	H-N(4)	H-C_β(7)	2.30	**3.98**	-0.29
25	H-C_β(4)	H_{ax}-C_α(1)	1.67	3.40	-0.22
26	H-N(5)	H-N(4)	0.11	-0.17	-0.14
27	H-N(5)	H_{ax}-C_α(4)	0.86	0.13	-0.03
28	H-N(5)	Me-C_α(4)	**-1.15**	-0.67	-0.45
29	H-N(5)	H-C_β(5)	-0.60	-0.63	-0.59
30	H-N(5)	H_{ax}-C_α(5)	0.06	0.19	-0.06
31	H-N(5)	H-N(6)	0.12	0.45	0.05
32	H-C_β(5)	H_{ax}-C_α(2)	2.00	3.56	0.03
33	H-C_β(5)	H-C_γ(5)	0.01	0.11	-0.06
34	H-C_β(5)	H_{eq}-C_α(5)	0.05	0.27	-0.05
35	H-N(6)	H-C_β(6)	-0.02	-0.04	0.00
36	H-N(6)	H_{ax}-C_α(6)	0.15	0.07	-0.05
37	H-N(6)	H_{ax}-C_α(5)	0.26	0.23	-0.01
38	H-C_β(6)	H_{ax}-C_α(3)	1.88	2.98	-0.17
39	H-C_β(6)	H_{eq}-C_α(6)	0.20	0.17	-0.11
40	H-N(7)	H_{ax}-C_α(6)	0.18	0.06	-0.11
41	H-N(7)	H-C_β(7)	-0.09	-0.13	-0.14
42	H-N(7)	H_{ax}-C_α(7)	-0.03	0.05	-0.10

Table S4: NOE distance violations calculated from the MD trajectories of the "VALXVAL" β-heptapeptide. The largest positive and negative violations are marked in boldface.

	H atoms		NOE distance violations (Å) FFc36	FFZhu	FFnew
1	H-N(1)	H-C_β(1)	-0.72	**-0.72**	**-0.72**
2	H-C_β(1)	H_{ax}-C_α(1)	-0.32	-0.25	-0.00
3	H-C_β(1)	H_{eq}-C_α(1)	-0.14	-0.13	-0.40
4	H-N(2)	H_{ax}-C_α(1)	0.31	0.17	-0.04
5	H-N(2)	H_{eq}-C_α(1)	-0.53	-0.39	-0.22
6	H-N(2)	H-C_β(2)	-0.40	-0.45	-0.41
7	H-N(2)	H-C_β(4)	3.15	3.93	0.19
8	H-N(2)	H-C_β(5)	**3.62**	**4.74**	0.07
9	H-C_β(2)	H-C_γ(2)	-0.03	-0.04	-0.03
10	H-C_β(2)	H_{eq}-C_α(2)	0.42	0.46	0.18
11	H-N(3)	H_{ax}-C_α(2)	0.59	0.31	0.03
12	H-N(3)	H_{eq}-C_α(2)	-0.65	-0.40	-0.21
13	H-N(3)	H-C_β(3)	-0.18	-0.24	-0.20
14	H-N(3)	H_{ax}-C_α(3)	0.03	0.10	-0.07
15	H-N(3)	H-N(4)	-0.09	0.15	-0.21
16	H-N(3)	H-C_β(5)	3.04	3.68	0.15
17	H-N(3)	H-C_β(6)	2.90	4.06	0.13
18	H-C_β(3)	H-C_δ(3)	0.08	0.04	0.08
19	H-N(4)	H_{ax}-C_α(3)	0.49	0.21	-0.09
20	H-N(4)	H_{eq}-C_α(3)	-0.08	0.03	0.12
21	H-N(4)	H-C_β(4)	0.00	-0.03	0.01
22	H-N(4)	H-C_γ(4)	-0.48	-0.55	-0.49
23	H-N(4)	H-C_β(6)	2.09	3.11	**0.27**
24	H-N(4)	H-C_β(7)	3.26	4.53	-0.13
25	H-C_β(4)	H_{ax}-C_α(1)	2.68	4.25	-0.07
26	H-N(5)	H-N(4)	-0.20	-0.10	-0.07
27	H-N(5)	H_{ax}-C_α(4)	0.19	0.15	-0.02
28	H-N(5)	Me-C_α(4)	**-0.74**	-0.69	-0.47
29	H-N(5)	H-C_β(5)	-0.58	-0.66	-0.62
30	H-N(5)	H_{ax}-C_α(5)	0.05	0.14	-0.00
31	H-N(5)	H-N(6)	0.17	0.33	0.12
32	H-C_β(5)	H_{ax}-C_α(2)	3.04	4.36	0.22
33	H-C_β(5)	H-C_γ(5)	-0.04	0.05	0.02
34	H-C_β(5)	H_{eq}-C_α(5)	0.05	0.23	-0.04
35	H-N(6)	H-C_β(6)	0.00	-0.05	-0.01
36	H-N(6)	H_{ax}-C_α(6)	0.20	0.10	0.01
37	H-N(6)	H_{ax}-C_α(5)	0.57	0.24	0.02
38	H-C_β(6)	H_{ax}-C_α(3)	2.04	3.39	-0.04
39	H-C_β(6)	H_{eq}-C_α(6)	0.09	0.18	-0.06
40	H-N(7)	H_{ax}-C_α(6)	0.33	0.09	-0.05
41	H-N(7)	H-C_β(7)	-0.08	-0.14	-0.14
42	H-N(7)	H_{ax}-C_α(7)	-0.01	0.15	-0.03

Table S5: NOE distance violations calculated from the MD trajectories of the “VALXVAL” β-heptapeptide when started from a fully extended conformation. The largest positive and negative violations are marked in boldface.

	H atoms		NOE distance violations (Å)		
			FFc36	FFZhu	FFnew
1	H-N(2)	H-CMe_2(2)	-0.49	-0.74	-0.26
2	H-N(2)	H-C_α(2)	-0.62	-0.17	-0.70
3	H-N(2)	H-C_β(1)	-1.11	-1.10	-0.11
4	H-N(2)	H-C_α(1)	-0.67	-0.66	-0.81
5	H_S-C_β(3)	H-CMe_2(3)	-0.50	-0.70	-0.59
6	H_S-C_β(3)	H-C_α(3)	-1.03	-0.93	-1.00
7	H-N(3)	H-C_β(2)	-0.60	-0.89	**0.10**
8	H-N(3)	H-C_α(2)	-0.65	-0.28	-0.79
9	H-N(4)	H_S-C_α(4)	-1.30	-1.25	-1.27
10	H-N(4)	H_R-C_α(4)	-0.18	-0.22	-0.08
11	H-N(5)	Me-C_α(5)	**-1.93**	**-2.14**	**-1.93**
12	H-N(5)	Me-C_β(5)	-1.02	-0.99	-0.97
13	H-N(5)	H_S-C_α(4)	-1.03	-0.60	-1.06
14	H-N(5)	H-C_α(5)	-0.88	-0.67	-0.82
15	H-N(5)	H-C_β(4)	-0.47	-0.75	-0.03
16	H-N(6)	H-C_α(6)	-0.55	-0.49	-0.50
17	H-N(6)	H-CMe_2(6)	-1.04	-1.06	-1.07
18	H-C_α(1)	H-C_β(6)	**0.38**	**4.43**	-0.45
19	H-C_α(2)	H-C_β(5)	-0.06	2.10	-0.20
20	Me_2-CH(2)	Me-C_α(5)	-1.02	-0.42	-1.35

Table S6: NOE distance violations calculated from the MD trajectories of the "HP6" β-hexapeptide. The largest positive and negative violations are marked in boldface.

References

Beke, Tamás, Csaba Somlai, and András Perczel (2006). "Toward a rational design of β-peptide structures". In: *Journal of computational chemistry* 27.1, pp. 20–38. URL: http://onlinelibrary.wiley.com/doi/10.1002/jcc.20299/full (visited on 02/10/2017).

Huang, Wei, Zhixiong Lin, and Wilfred F. van Gunsteren (May 2011). "Validation of the GROMOS 54A7 Force Field with Respect to β-Peptide Folding". In: *Journal of Chemical Theory and Computation* 7.5, pp. 1237–1243. ISSN: 1549-9618. DOI: 10.1021/ct100747y. URL: http://dx.doi.org/10.1021/ct100747y (visited on 01/09/2017).

Schrödinger, LLC (Nov. 2015). *The PyMOL Molecular Graphics System, Version 1.8.*

Zhu, Xiao et al. (2010). "Establishing effective simulation protocols for β- and α/β-peptides. III. Molecular mechanical model for acyclic β-amino acids". en. In: *Journal of Computational Chemistry*, NA–NA. ISSN: 01928651, 1096987X. DOI: 10.1002/jcc.21493. URL: http://doi.wiley.com/10.1002/jcc.21493 (visited on 01/16/2017).
